# RPM-1 Uses Both Ubiquitin Ligase and Phosphatase-Based Mechanisms to Regulate DLK-1 during Neuronal Development

**DOI:** 10.1371/journal.pgen.1004297

**Published:** 2014-05-08

**Authors:** Scott T. Baker, Karla J. Opperman, Erik D. Tulgren, Shane M. Turgeon, Willy Bienvenut, Brock Grill

**Affiliations:** 1 Department of Neuroscience, The Scripps Research Institute, Jupiter, Florida, United States of America; 2 Department of Pharmacology, University of Minnesota, Minneapolis, Minnesota, United States of America; 3 CNRS, Campus de Recherche de Gif, Gif-sur-Yvette, France; Stanford University School of Medicine, United States of America

## Abstract

The Pam/Highwire/RPM-1 (PHR) proteins are key regulators of neuronal development that function in axon extension and guidance, termination of axon outgrowth, and synapse formation. Outside of development, the PHR proteins also regulate axon regeneration and Wallerian degeneration. The PHR proteins function in part by acting as ubiquitin ligases that degrade the Dual Leucine zipper-bearing Kinase (DLK). Here, we show that the *Caenorhabditis elegans* PHR protein, Regulator of Presynaptic Morphology 1 (RPM-1), also utilizes a phosphatase-based mechanism to regulate DLK-1. Using mass spectrometry, we identified Protein Phosphatase Magnesium/Manganese dependent 2 (PPM-2) as a novel RPM-1 binding protein. Genetic, transgenic, and biochemical studies indicated that PPM-2 functions coordinately with the ubiquitin ligase activity of RPM-1 and the F-box protein FSN-1 to negatively regulate DLK-1. PPM-2 acts on S874 of DLK-1, a residue implicated in regulation of DLK-1 binding to a short, inhibitory isoform of DLK-1 (DLK-1S). Our study demonstrates that PHR proteins function through both phosphatase and ubiquitin ligase mechanisms to inhibit DLK. Thus, PHR proteins are potentially more accurate and sensitive regulators of DLK than originally thought. Our results also highlight an important and expanding role for the PP2C phosphatase family in neuronal development.

## Author Summary

The molecular mechanisms that govern formation of functional synaptic connections are central to brain development and function. We have used the nematode *C. elegans* to explore the mechanism of how the intracellular signaling protein RPM-1 regulates neuronal development. Using a combination of proteomic, genetic, transgenic, and biochemical approaches we have shown that RPM-1 functions through a PP2C phosphatase, PPM-2, to regulate the activity of a MAP3 kinase, DLK-1. Our results indicate that a combination of PPM-2 phosphatase activity and RPM-1 ubiquitin ligase activity inhibit DLK-1.

## Introduction

As a neuron develops, its axon must execute several important tasks. Initially, the axon extends toward target cells by sensing extracellular guidance cues. Upon reaching its target, the axon detects guidepost signals and makes presynaptic connections with its postsynaptic partner(s) to form synapses [1]–[3]. At some point during these events, an axon must terminate outgrowth in a spatially and anatomically accurate manner. At present, we know little about how axon termination is governed, and even less about how this process is integrated with synapse formation.

While axon extension, synapse formation, and termination of axon outgrowth are often analyzed individually, there is substantial evidence suggesting that these events are coordinated and integrated during development. Elegant live imaging studies using developing retinal ganglion cells (RGC) in Xenopus and zebrafish showed there is a tight temporal and spatial link between axon outgrowth and synapse formation [4]–[7]. In cultured neurons, synaptogenesis proceeds rapidly upon contact between an actively growing axon and its dendritic partner [8], [9]. At the Drosophila neuromuscular junction (NMJ), synapse formation displays tight temporal linkage with axon outgrowth [10], and new synapses are formed at the terminal tips of growing axons [11]. Studies in *C. elegans*, Drosophila, and zebrafish have also shown a link between synaptic activity, and axon outgrowth and branching [5], [12]–[14]. Finally, studies in invertebrate and vertebrate systems have shown that morphogens, axon guidance cues, and cell adhesion molecules function in both axon outgrowth and synapse formation [15]. This functional promiscuity is likely to reflect, in part, that extracellular signals take on different roles during development, and in different types of neurons. However, some of these observations also highlight a potential role for extracellular signals in coordinating axon outgrowth and termination with synapse formation. Two cases of particular note from *C. elegans* where guidance cues regulate both axon guidance and synapse formation in a single type of neuron are the role of UNC-6 (Netrin) in the RIA and AIY neurons [16], and studies on UNC-6 and Wnts on the DA9 neuron [17]–[19].

At present, the identity of intracellular signaling proteins that may coordinate axon outgrowth, synapse formation, and termination of outgrowth remain unclear. Nonetheless, such coordinators would be likely to meet several criteria. 1) They would need to be evolutionarily conserved, and function in multiple events during the development of an individual neuron. 2) They would need to be signaling molecules that function intracellularly and cell autonomously. 3) Their activity would need to be regulated by upstream, presumably extracellular, signals. 4) They would need to regulate multiple downstream signaling pathways, and do so both positively and negatively. 5) They would need to regulate downstream signaling pathways in a relatively precise and accurate manner, most likely to control both gene transcription and short-term/local signaling events.

Members of the PHR protein family meet several of these criteria, and have been put forward as candidate molecules that may coordinate different events during neuronal development [20], [21]. The PHR proteins are highly conserved with a single family-member in worms, flies, fish, mice, and humans [21]. The PHR proteins function in a range of developmental events playing roles in axon extension [22]–[24], axon guidance [20], [23], [25]–[28], axon termination [22], [29], [30], and synapse formation [24], [29], [31], [32]. Importantly, studies on the mechanosensory neurons of *C. elegans* and the motor neurons of Drosophila have shown that the PHR proteins function cell autonomously to regulate synapse formation, as well as axon termination and branching in individual neurons [29], [32]. The functional importance of the PHR protein family is further highlighted by studies showing that they regulate axon regeneration [33], [34], axon degeneration [35], [36], and aversive long-term memory [37].

While our picture of the mechanism for PHR protein function is incomplete, previous studies have established that PHR proteins regulate multiple intracellular signaling pathways both negatively and positively [22], [38]–[46]. Through these signaling mechanisms, the PHR proteins function in late endosome/lysosome trafficking or formation [39], receptor trafficking and endocytosis [20], [47], [48], microtubule dynamics [22], [26], and gene transcription [38], [41], [46], [49]. The human PHR protein called Protein Associated with Myc (Pam) or Myc binding protein 2 (MYCBP2) also binds to F-actin [50], which suggests a further link between the PHR proteins and the cytoskeleton. While our knowledge of how the PHR proteins function has grown rapidly, it remains unclear if the PHR proteins have the potential to meet two important criteria as candidate coordinators of neuronal development. Are the PHR proteins regulated by upstream signals that are triggered by extracellular guidance cues, adhesion molecules, or morphogens? Do the PHR proteins have the potential to precisely and accurately control the signaling pathways they regulate?

An important, conserved function of the PHR proteins is to ubiquitinate and negatively regulate MAP kinase kinase kinase (MAP3K) signaling. The ubiquitin ligase activity of the PHR proteins requires an F-box protein called F-box Synaptic protein 1 (FSN-1) in *C. elegans* and Drosophila [51], [52] and Fbxo45 in mice [53]. One MAP3K target of the PHR proteins is DLK [22], [38], [41], [54]. Mammalian DLK (also called MAP3K12) has functional orthologs in *C. elegans* (DLK-1), and Drosophila (Wallenda). PHR proteins also use ubiquitination to negatively regulate the TSC complex [46], [49] and NMNAT [35], [36].

DLK plays an important and conserved function in neuronal development by regulating synapse formation, axon extension, and termination of axon outgrowth [38], [39], [41], [55]–[57]. In mature neurons, DLK is critical for axon regeneration [33], [34], [40], [58]–[60], and also functions in axon degeneration [61], [62].

Progress has been made in understanding how DLK is regulated. Studies using cell lines and *in vitro* biochemical methods have shown that DLK is phosphorylated, self-dimerizes, and autophosphorylates [63]–[65]. JNK Interacting Proteins (JIP) bind to DLK potentially acting as scaffolds to regulate DLK activity or localization [62], [65]. In Drosophila, the cytoskeletal regulatory protein Spectraplakin inhibits DLK [66]. Pharmacological studies have suggested that the phosphatase calcineurin [63] and the kinase Src [64] may regulate DLK, but evidence that these molecules regulate DLK in neurons is absent. Finally, work in *C. elegans* has shown that calcium signaling functions upstream of DLK-1 [67] to regulate binding of a short, inhibitory isoform of DLK-1 (DLK-1S) to full length DLK-1 (DLK-1L) [68].

Here, we show that RPM-1 (the *C. elegans* PHR protein) employs a phosphatase-based mechanism to inhibit DLK-1. Using a combination of proteomics and genetics, we show that RPM-1 binds to and positively regulates Protein Phosphatase Magnesium/Manganese dependent 2 (PPM-2), a member of the PP2C phosphatase family. We provide genetic, transgenic, and biochemical evidence showing that PPM-2 negatively regulates DLK-1L by direct dephosphorylation at a specific serine residue that is implicated in binding to DLK-1S. Our findings demonstrate that RPM-1 harnesses two independent mechanisms to negatively regulate DLK-1, ubiquitination and PPM-2 phosphatase activity. These results suggest that RPM-1 may function through ubiquitin ligase activity to regulate long-term DLK-1 signaling and through the phosphatase PPM-2 to regulate short-term/local DLK-1 signaling. Thus, PHR proteins may be more accurate and precise regulators of DLK than originally thought.

## Results

### Identification of PPM-2 as an RPM-1 binding protein

To better understand the mechanism of how RPM-1 regulates neuronal development, we previously purified RPM-1 and used mass spectrometry to identify RPM-1 binding proteins [39]. In brief, a fusion protein of RPM-1 and GFP (RPM-1::GFP) was transgenically expressed in *C. elegans* using the native *rpm-1* promoter, which is expressed in neurons. An anti-GFP antibody was used to immunoprecipitate RPM-1::GFP from whole worm lysates, and RPM-1 binding proteins were identified using mass spectrometry and *de novo* peptide sequencing. Using this approach, we previously identified GLO-4 and RAE-1 as functional RPM-1 binding proteins [39], [45]. This proteomic screen also identified a phosphatase, PPM-2 (*T23F11.1*).

PPM-2 is in the Protein Phosphatase 2C (PP2C) family, which is also called the Protein Phosphatase Mg/Mn dependent (PPM) family. PP2C phosphatases are single subunit enzymes with serine/threonine phosphatase activity. Previous studies have shown that PPM-2 (also called PP2Cγ2) has orthologs in yeast and Drosophila [69]. We have found evidence of a PPM-2 ortholog in the genome sequence of the protochordate *Ciona intestinalis* (XP_002127931.1).

**Figure 1:**
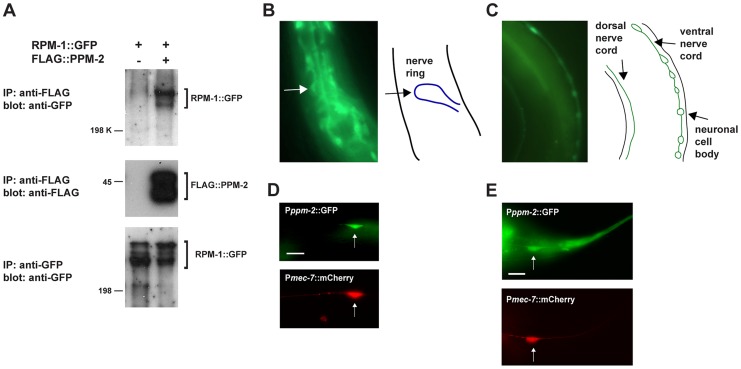
RPM-1 binds to the PP2C phosphatase PPM-2. (*A*) RPM-1::GFP was transgenically expressed in the neurons of *C. elegans* alone or in combination with FLAG::PPM-2. Coprecipitating RPM-1::GFP was detected with FLAG::PPM-2 (upper panel). Levels of FLAG::PPM-2 (middle blot) and RPM-1::GFP (lower blot) were determined by immunoprecipitation (IP). (*B and C*) At left are epifluorescent images of transgenic animals expressing GFP from the native *ppm-2* promoter (P*ppm-2*::GFP). At right are schematic diagrams of the cells, nerve cords or regions of interest. *Pppm-2::GFP* expression was detected in (*B*) the nerve ring, and in (*C*) the dorsal and ventral nerve cords. (*D and E*) Shown are epifluorescent images of transgenic animals expressing both GFP from the native *ppm-2* promoter (P*ppm-2*::GFP) and mCherry from a cell-specific promoter for the mechanosensory neurons (P*mec-*7::mCherry). Expression of P*ppm-2*::GFP detected in (*D*) an ALM mechanosensory neuron (arrow) and (*E*) a PLM mechanosensory neuron (arrow). In all cases, multiple independently derived transgenic lines showed similar results, and images from a representative transgenic line are shown. Scale bars are 10 µm.

Our proteomic analysis identified 5 unique peptides that covered 15% of the total PPM-2 protein sequence (Figure S1). To confirm our proteomic result, we performed coimmunoprecipitation (coIP) from transgenic *C. elegans*. Animals were generated that expressed FLAG epitope tagged PPM-2 (FLAG::PPM-2) and RPM-1::GFP specifically in neurons using a pan-neuronal promoter (P*rgef-1*) and the *rpm-1* promoter, respectively. FLAG::PPM-2 was immunoprecipitated from whole worm lysates using an anti-FLAG antibody, and coprecipitating RPM-1::GFP was detected in immunoblots with an anti-GFP antibody (Figure 1A). This observation confirmed that PPM-2 is part of a neuronal protein complex that includes RPM-1.

### *ppm-2* regulates axon termination in the mechanosensory neurons

**Figure 2:**
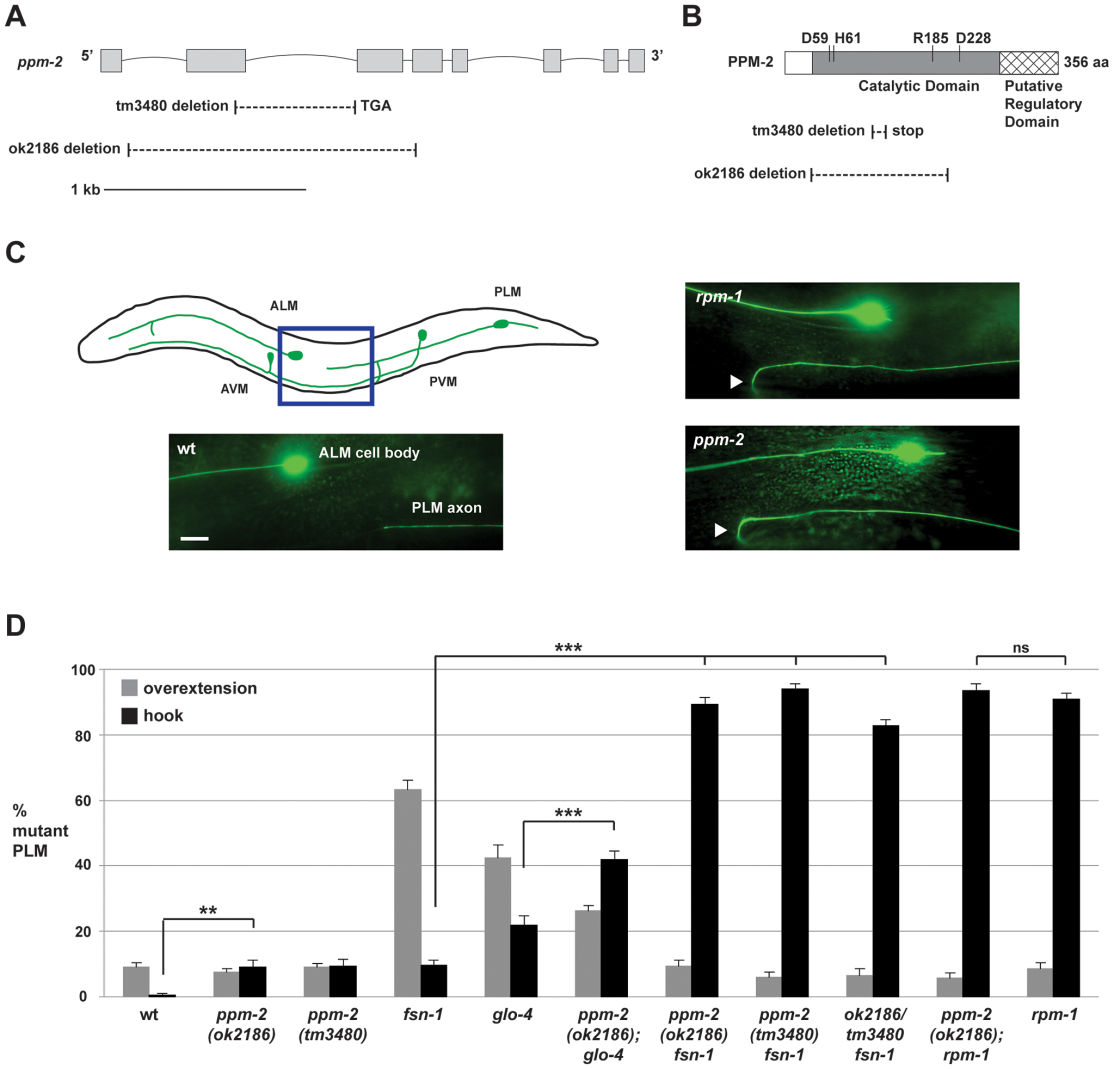
*ppm-2* regulates axon termination of PLM neurons. (*A*) Schematic diagram of the *ppm-2* open reading frame. Exons are shown with grey boxes and introns as lines. Deletions generated by *ok2186* and *tm3480* are shown below. (*B*) Schematic diagram of the PPM-2 protein. Conserved residues that are required for catalytic activity are highlighted. Protein sequence deleted by *ok2186* and *tm3480* are shown below. (*C and D*) Defects in axon termination of the PLM mechanosensory neurons were visualized using *muIs32* (P*_mec-7_*GFP). (*C*) Upper panel is a schematic diagram showing the mechanosensory neurons of *C. elegans* (modified from Worm Atlas). The blue box highlights the region shown below that was visualized using epifluorescent microscopy. An example of a PLM axon that overextends and hooks (hook) is shown for both *ppm-2(ok2186)-/-* and *rpm-1-/-* genotypes (arrowheads). Scale bar is 10 µm. (*D*) Quantitation of axon termination defects (hook represented in black, or overextension alone represented in grey) for the indicated genotypes. Averages are shown for data collected from 5–8 independent counts of 20–30 PLM neurons from young adult worms (16–20 hours post L4) grown at 23°C. Error bars represent the standard error of the mean, and significance was determined using an unpaired *t*-test. **p<0.01, ***p<0.001 and ns  =  not significant.

Our promoter expression studies showed that the *ppm-2* promoter is active in many neurons including those of the nerve ring (Figure 1B), the motor neurons (Figure 1C), and the mechanosensory neurons (Figure 1D and E). *ppm-2* promoter activity was also detected in gut, muscle, and pharynx (data not shown). Because PPM-2 binds to RPM-1 and is expressed in a wide range of neurons, we hypothesized that PPM-2 might function in neuronal development, similar to RPM-1. To test this hypothesis, we analyzed two alleles of *ppm-2*, *ok2186* and *tm3480*. As shown in Figure 2A, *ok2186* is a deletion mutation that removes 1421 base pairs of sequence. *ok2186* deletes 5′ intronic sequence as well as exonic sequence. This suggests that *ok2186* results in a truncated protein at minimum and presumably causes a premature stop. Importantly, *ok2186* deletes three conserved residues (D59, H61 and R185, Figure 2B) that are required for the catalytic activity of PP2C phosphatases [70], [71]. Thus, *ok2186* is likely to be a molecular null allele. *tm3480* is a deletion that removes 594 base pairs of *ppm-2* sequence, generates a frame-shift, and results in nonsense sequence encoding 7 amino acids prior to premature termination of the open reading frame (Figure 2A). The premature stop in *tm3480* leads to a predicted protein that is truncated and lacks two conserved residues (R185 and D228) that are required for catalytic activity (Figure 2B) [70], [71]. Therefore, *tm3480* is likely to be a molecular null allele. On gross examination, *ppm-2-/-* mutants had normal body size and shape, and moved normally.

Previous studies in *C. elegans* have shown that *rpm-1* regulates axon termination in the mechanosensory neurons (touch receptor neurons), which sense soft touch [29], [39]. These neurons are an excellent model to study axon termination for two reasons. First, *C. elegans* mechanosensory neurons terminate axon extension at precise anatomical locations [72], [73], which are easily visualized using a transgene (*muIs32*) that expresses GFP in these cells [74]. Second, *C. elegans* and mammals share a similar but poorly understood mechanism that governs axon termination of sensory neurons. This is supported by the observation that loss-of-function mutations in *C. elegans rpm-1*, Drosophila *Highwire*, and murine Phr1 result in axon termination defects in sensory neurons [22], [29], [30].

*C. elegans* has two Posterior Lateral Microtubule (PLM) mechanosensory neurons. Each PLM neuron extends a single axon that terminates extension prior to the cell body of the Anterior Lateral Microtubule (ALM) mechanosensory neuron (Figure 2C, see schematic). In contrast, in *rpm-1*-/- animals the PLM axons fail to terminate extension properly, overgrow, and hook toward the ventral side of the animal (Figure 2C). These overextension and hook defects, which will be referred to as “hook” defects for brevity of presentation, are highly penetrant in *rpm-1-/-* mutants (90.8+/−2.0% hook, Figure 2D). As noted in previous work, *rpm-1-/-* mutants also have a lower penetrance defect in which the PLM axon overextends, but does not hook (Figure 2D) [45], [75]. We refer to this phenotype simply as an “overextension” defect. *ppm-2(ok2186)-/-* and *ppm-2(tm3480)-/-* animals had hook defects that were of similar severity to *rpm-1-/-* mutants (Figure 2C), but the defects occurred with much lower penetrance (compare 9.0+/−2.2% for *ppm-2(ok2186)* and 9.3+/−2.2% for *ppm-2(tm3480)* to 90.8+/−2.0% for *rpm-1*, Figure 2D). *ppm-2-/-* mutants did not show significant overextension defects (Figure 2D).

In order to understand the genetic relationship between PPM-2 and other RPM-1 binding proteins, we made double mutants of *ppm-2* with *fsn-1* or *glo-4*. FSN-1 is an F-box protein that mediates the ubiquitin ligase activity of RPM-1 [39], [51], and GLO-4 is a putative guanine nucleotide exchange factor (GEF) that is positively regulated by RPM-1 [39]. Previous studies established that *fsn-1* and *glo-4* function in parallel genetic pathways [39], [75]. However, both *fsn-1* and *glo-4* function downstream of *rpm-1*, and in the same pathway as *rpm-1* to regulate axon termination and synapse formation [39], [51]. Consistent with prior work, *fsn-1-/-* and *glo-4-/-* single mutants displayed hook defects, but these defects occurred with less penetrance than in *rpm-1-/-* animals (compare 22.0+/−2.7% for *glo-4* and 9.5+/−1.6% for *fsn-1* to 90.8+/−2.0% for *rpm-1*, Figure 2D). In contrast, *ppm-2-/- fsn-1-/-* double mutants had strongly enhanced penetrance of hook defects (compare 89.4+/−2.0% for *ppm-2(ok2186) fsn-1* and 94.0+/−1.5% for *ppm-2(tm3480) fsn-1* to 9.5+/−1.6% for *fsn-1*, Figure 2D). Hook defects were also mildly enhanced in *ppm-2-/-; glo-4-/-* double mutants (compare 41.8+/−2.8% for *ppm-2(ok2186); glo-4* to 22.0+/−2.7% for *glo-4*, Figure 2D).

Aside from defective axon termination in the PLM neurons, *rpm-1-/-* mutants also had other defects in the mechanosensory neurons, similar to what was described previously [29], [39]. In *rpm-1-/-* mutants, 85.6+/−1.6% of the PLM neurons that were analyzed lacked a synaptic branch (Figure S2). Notably, a previous study showed that the absence of the PLM synaptic branch was due to a failure in synapse formation and/or maturation [29]. *ppm-2-/- fsn-1-/-* double mutants had enhanced penetrance of defects in synaptic branch extension (70.5+/−4.3% for *ppm-2(ok2186) fsn-1*) compared to *ppm-2-/-* single mutants (6.6+/−1.3% for *ppm-2(ok2186)*) or *fsn-1-/-* single mutants (3.5+/−1.2%) (Figure S2). Thus, *ppm-2-/- fsn-1-/-* double mutants have enhanced defects in both axon termination and synaptogenesis in PLM neurons.

In *rpm-1-/-* animals, 66.1+/−2.1% of the ALM neurons had axon termination defects in which the ALM axon overgrew and extended towards the posterior of the animal, which we refer to as big hooks (Figure S3). *ppm-2-/- fsn-1-/-* double mutants had enhanced penetrance of defects in axon termination of the ALM neurons (37.6+/−4.1% big hook for *ppm-2(ok2186) fsn-1*) compared to either single mutant (0% for *ppm-2(ok2186)* and 12.5+/−2.0% for *fsn-1*, Figure S3).

While both alleles of *ppm-2* are likely to be molecular null alleles, we wanted to confirm this experimentally. To do so, we generated *ppm-2(ok2186/tm3480) fsn-1-/-* transheterozygous animals. The penetrance of hook defects in the PLM neurons was enhanced in *ppm-2(ok2186/tm3480) fsn-1-/-* animals (82.8+/−2.0%) compared to *fsn-1-/-* single mutants (9.5+/−1.6%) and *ppm-2-/-* single mutants (9.0+/−2.2% for *ppm-2(ok2186)* and 9.3+/−2.2% for *ppm-2(tm3480)*, Figure 2D). These results are consistent with the interpretation that both alleles of *ppm-2* are genetic nulls. Thus, our observation that null alleles of *ppm-2* enhance null alleles of *glo-4* or *fsn-1* is consistent with *ppm-2* functioning in a parallel genetic pathway to both *glo-4* and *fsn-1*.

Previous studies have shown that *rpm-1*-/- mutant phenotypes are partially suppressed by loss-of-function (lf) mutations in *dlk-1*, because DLK-1 is targeted for ubiquitination and degradation by RPM-1 [38], [39]. Given that PPM-2 is an RPM-1 binding protein, it was possible that RPM-1 might ubiquitinate and degrade PPM-2. To test this possibility, we constructed *ppm-2-/-; rpm-1-/-* double mutants. The PLM and ALM axon termination defects (Figure 2B and S3), and the defects in synaptic branch extension of the PLM neurons (Figure S2) were similar between *ppm-2-/-; rpm-1-/-* double mutants and *rpm-1-/-* single mutants. These observations suggest that PPM-2 is not targeted for ubiquitination and degradation by RPM-1. These results are also consistent with *ppm-2* functioning in the same genetic pathway as *rpm-1*.

It should be noted that although *rpm-1-/-* mutants have defects in axon termination and synapse formation in the mechanosensory neurons, previous studies have shown that *rpm-1-/-* mutants have normal locomotion, have normal soft touch sensation and are mildly dumpy [29], [31]. We observed that *ppm-2-/-* mutants had normal body size, normal locomotion, and sensed soft touch normally (data not shown). Thus, defects in axon termination associated with loss of function in the RPM-1 pathway do not lead to changes in the ability of the mechanosensory neurons to sense touch.

### *ppm-2* functions cell autonomously, downstream of *rpm-1* to regulate axon termination

**Figure 3:**
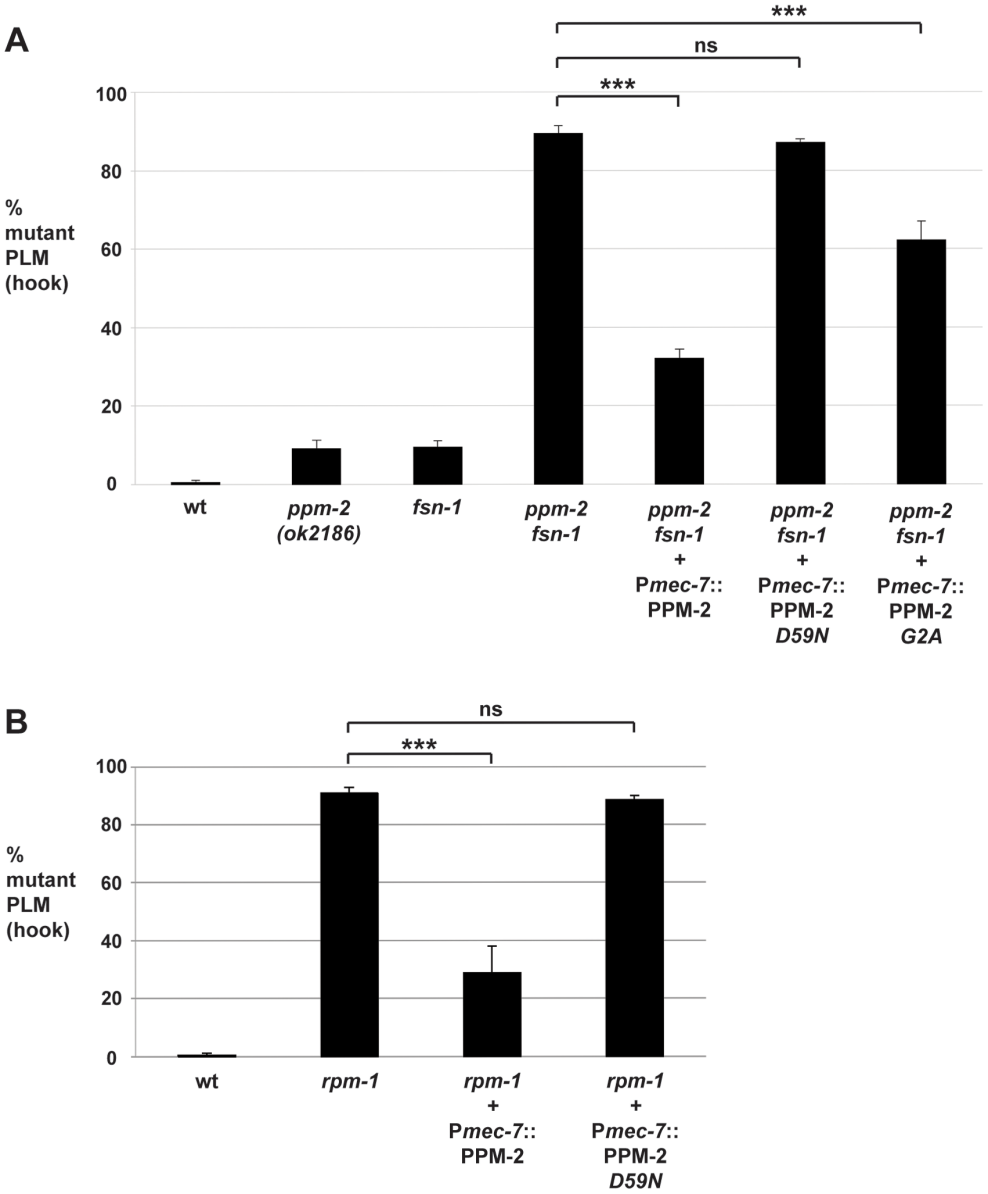
*ppm-2* functions cell autonomously downstream of *rpm-1*. The PLM axon termination defects (hook) were quantified for all genotypes shown using the transgene *muIs32*. (*A*) A cell specific promoter (P*mec-7*) was used to transgenically express wild-type PPM-2, phosphatase-dead PPM-2 *D59N*, or PPM-2 *G2A* that was not N-myristoylated in the PLM neurons of *ppm-2-/- fsn-1-/-* double mutants. (*B*) A cell specific promoter (P*mec-7*) was used to transgenically express PPM-2 or phosphatase-dead PPM-2 *D59N* in *rpm-1-/-* single mutants. Averages are shown for data collected from 5 or more transgenic lines for each genotype. In all experiments, young adult worms (16–20 hours post L4) grown at 23°C were analyzed. Error bars represent the standard error of the mean, and significance was determined using an unpaired *t*-test. ***p<0.001 and ns  =  not significant.

Having established that *ppm-2* regulates axon termination in the mechanosensory neurons, we next sought to determine if the genetic lesion in *ppm-2(ok2186)* was responsible for the observed axon termination defects. Given that RPM-1 and its known binding proteins function cell autonomously in the mechanosensory neurons [29], [39], [45], we also wanted to determine if PPM-2 functions cell autonomously. Using a transgenic approach, *ppm-2-/- fsn-1-/-* double mutants were engineered with an extrachromosomal array expressing PPM-2. We opted to analyze transgenic rescue using *ppm-2-/- fsn-1-/-* double mutants because axon termination defects were more penetrant in these animals than *ppm-2-/-* single mutants, thus facilitating ease of analysis. When PPM-2 was expressed in the mechanosensory neurons using a cell specific promoter (P*mec-7*), the axon termination defects in *ppm-2-/- fsn-1-/-* double mutants were significantly rescued (compare 89.4+/−2.0% for *ppm-2 fsn-1* with 32.0+/−2.5% for *ppm-2 fsn-1 +* P*mec-7*::PPM-2, Figure 3A). In contrast, PPM-2 that was point mutated at a conserved residue (D59N) that is required for PP2C phosphatase activity, did not rescue the defects in *ppm-2-/- fsn-1-/-* double mutants (Figure 3A). These observations are consistent with *ppm-2* functioning cell autonomously through its phosphatase activity to regulate axon termination in the mechanosensory neurons.

Our genetic analysis showed that *ppm-2* functions in the same pathway as *rpm-1*, and we wanted to test if *ppm-2* functions up or downstream of *rpm-1*. To do so, we generated *rpm-1-/-* mutants that carried a transgenic extrachromosomal array that expressed PPM-2 specifically in the mechanosensory neurons. We observed that overexpression of PPM-2 partially rescued the axon termination defects in *rpm-1-/-* mutants (compare 90.8+/−2.0% for *rpm-1* to 28.8+/−9.2% for *rpm-1* + P*mec-7*::PPM-2, Figure 3B). Defects were not rescued by overexpression of PPM-2 *D59N* (Figure 3B). These observations are consistent with *ppm-2* functioning downstream of *rpm-1*.

### N-myristoylation is required for PPM-2 to be fully functional

During our mass spectrometry analysis, we detected N-myristoylation of a PPM-2 peptide (Figure S4A). Sequence analysis confirmed that the N-myristoylated glycine (amino acid 2) in PPM-2 is highly conserved with other PP2C family phosphatases (Figure S4B). To test if N-myristoylation was important for the function of PPM-2, we generated a point mutant of PPM-2, G2A, that cannot be myristoylated. We found that transgenic expression of PPM-2 *G2A* moderately rescued the axon termination defects in *ppm-2-/- fsn-1-/-* animals compared to wild-type PPM-2 (compare 89.4+/−2.0% for *ppm-2 fsn-1* to 32.0+/−2.5% for *ppm-2 fsn-1 +* P*mec-7*::PPM-2 and 62.2+/−4.8% for *ppm-2 fsn-1* + P*mec-7*::PPM-2 *G2A*, Figure 3A). This observation demonstrates that N-myristoylation is required for PPM-2 to function with full efficacy.

### PPM-2 negatively regulates DLK-1

**Figure 4:**
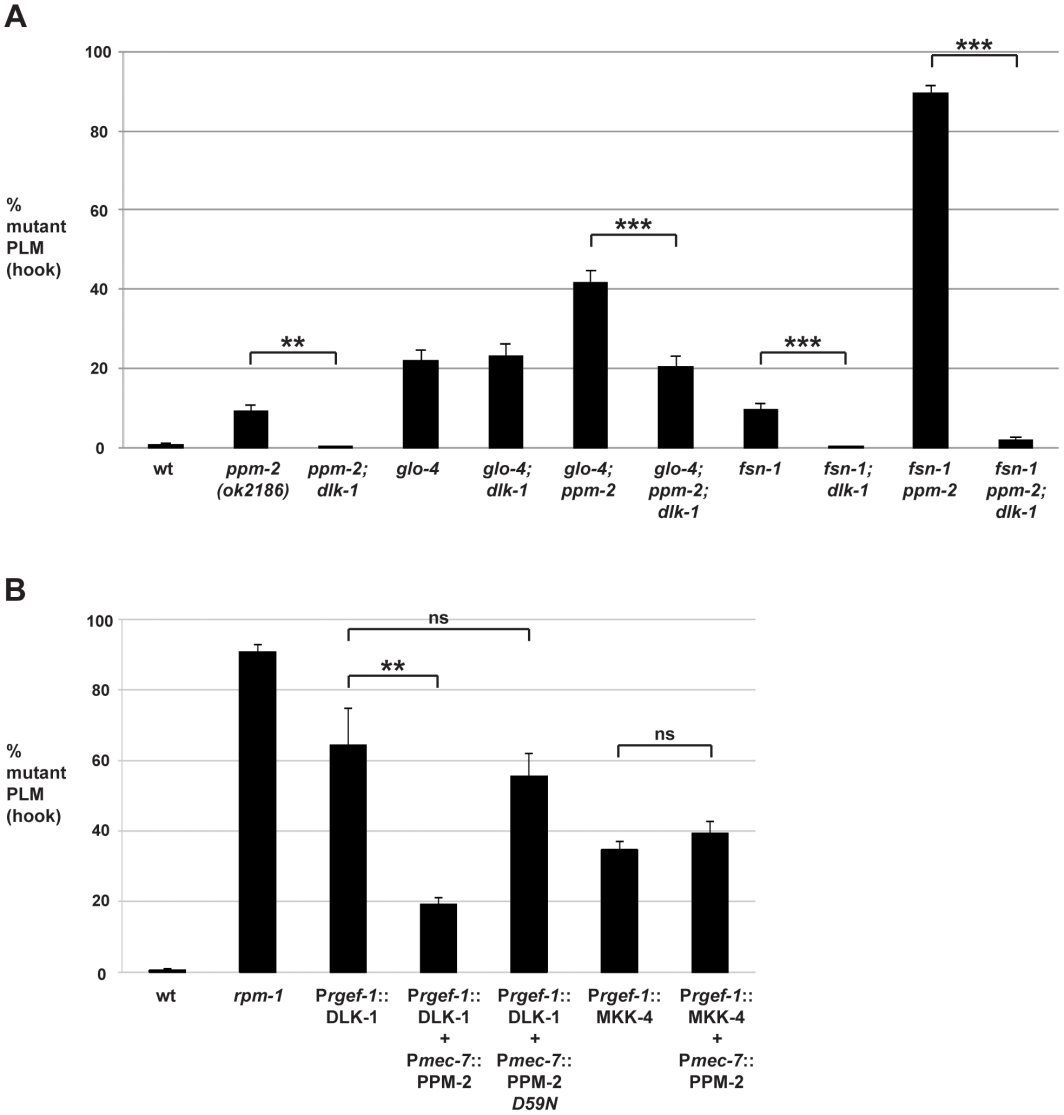
PPM-2 negatively regulates the MAP3K DLK-1. PLM axon termination defects (hook) were quantified for the indicated genotypes using the transgene *muIs32*. (*A*) Loss of function in *dlk-1* suppresses the axon termination defects in *ppm-2-/-* single mutants and *glo-4-/-; ppm-2-/-* double mutants. Shown are averages for data collected from 5–8 independent counts of 20–30 PLM neurons from young adult worms (16–20 hours post L4) grown at 23°C for each genotype. (*B*) Transgenic overexpression of the MAP3K DLK-1, or the MAP2K MKK-4 results in PLM axon termination defects (hook). Coexpression of PPM-2 rescues defects caused by overexpression of DLK-1, but not MKK-4. Shown are averages for data pooled from 5 or more transgenic lines for the indicated genotypes; young adult worms grown at 23°C were analyzed. For A and B, error bars represent the standard error of the mean, and significance was determined using an unpaired *t*-test. **p<0.005, ***p<0.001 and ns  =  not significant.

Previous studies in yeast, *Arabidopsis*, *C. elegans*, Drosophila, and cultured mammalian cells have shown that PP2C phosphatases can negatively regulate MAPK or MAP3K signaling [71], [75]–[79]. Given that RPM-1 negatively regulates a MAPK pathway that includes DLK-1, MKK-4 and PMK-3, we hypothesized that PPM-2 might also negatively regulate one or more of the kinases in this pathway. We began our analysis by studying the most upstream kinase in the pathway, the MAP3K DLK-1. Consistent with the interpretation that PPM-2 negatively regulates DLK-1, axon termination defects in the PLM neurons were suppressed in *ppm-2-/-; dlk-1-/-* double mutants (0% defect) compared to *ppm-2-/-* single mutants (9.0+/−2.2%, Figure 4A). Because PLM axon termination defects occur in *ppm-2-/-* mutants with low penetrance, we also performed our suppressor analysis using *glo-4-/-; ppm-2-/-* double mutants. Consistent with a previous study [39], we found that the hook defects in *glo-4-/-* single mutants were not suppressed by *dlk-1* (lf) (Figure 4A). In contrast, the enhanced penetrance of hook defects seen in *glo-4-/-; ppm-2-/-* double mutants (41.8+/−2.8%) was suppressed in *glo-4-/-; ppm-2-/-; dlk-1-/-* triple mutants (20.5+/−2.6%, Figure 4A).

Previous studies in flies have shown that DLK/Wallenda functions downstream of DFsn [52]. Consistent with these findings, we found that *fsn-1-/-; dlk-1-/-* double mutants were fully suppressed for hook defects compared to *fsn-1-/-* single mutants (compare 9.5+/−1.6% for *fsn-1* to 0% for *fsn-1; dlk-1*, Figure 4A). We also observed that enhanced hook defects in *fsn-1-/- ppm-2-/-* double mutants were fully suppressed in *fsn-1-/- ppm-2-/-; dlk-1-/-* triple mutants (compare 89.4+/−2.0% for *fsn-1 ppm-2* to 1.69+/−0.8% for *fsn-1 ppm-2; dlk-1*, Figure 4A). These results suggest that *ppm-2* and *fsn-1* function in a parallel genetic pathway that converges on the common target of *dlk-1*.

Having shown that PPM-2 negatively regulates the DLK-1 pathway, we next wanted to test if a specific kinase(s) in the pathway was regulated by PPM-2. To do so, we generated transgenic animals with extrachromosomal arrays that expressed a kinase in the DLK-1 pathway alone, or in combination with PPM-2. Similar to our previous observations [75], transgenic overexpression of DLK-1 or its downstream kinase MKK-4 using a pan-neuronal promoter (P*rgef-1*) resulted in axon termination defects in the PLM neurons (64.3+/−10.4% for P*rgef-1*::DLK-1 and 34.7+/−2.5% for P*rgef-1*::MKK-4, Figure 4B). Notably, MKK-4 overexpression did not cause as penetrant a phenotype as DLK-1 overexpression, presumably because the activity of MKK-4 is limited by the amount of endogenous upstream DLK-1 kinase activity. Coexpression of PPM-2 significantly rescued the defects caused by overexpression of DLK-1, but not the defects caused by overexpression of MKK-4 (compare 64.3+/−10.4% for P*rgef-1*::DLK-1 to 19.2+/−2.0% for P*rgef-1*::DLK-1 + P*mec-7*::PPM-2, Figure 4B). It should be noted that we expressed DLK-1 and MKK-4 at moderate levels (2.5 ng/µL PCR product injected for DLK-1 and 5 ng/µL for MKK-4) in order to maximize the potential for rescue by coexpression of PPM-2. Importantly, incorporation of a second transgene into arrays did not rescue defects caused by DLK-1 overexpression, as inactive PPM-2 *D59N* did not show rescue (Figure 4B). Thus, PPM-2 acts through its phosphatase activity to negatively regulate DLK-1, but not MKK-4. Because PMK-3 functions downstream of MKK-4 [38], our results are also consistent with the conclusion that PPM-2 does not regulate PMK-3. Overall, our results indicate that PPM-2 negatively regulates an upstream activator of DLK-1, acts on an inhibitor of DLK-1, or negatively regulates DLK-1 directly.

### PPM-2 acts on DLK-1

**Figure 5:**
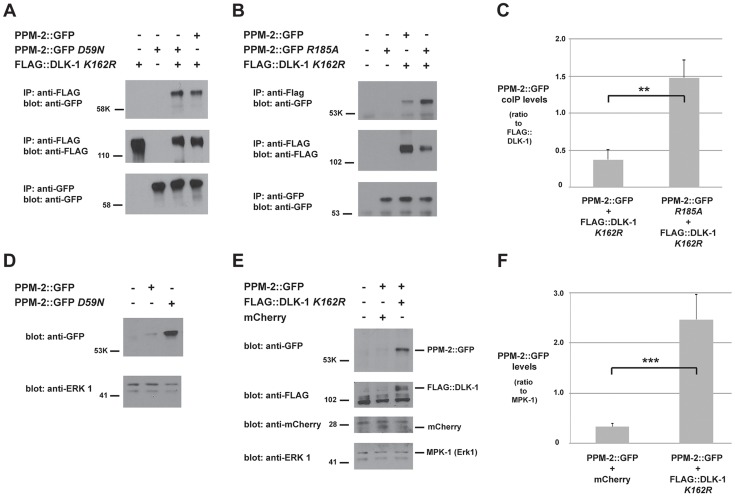
PPM-2 binds to DLK-1. CoIP from transgenic whole worm lysates showing that (*A*) PPM-2::GFP and PPM-2::GFP *D59N* bind to FLAG::DLK-1 *K162R* (upper panel) (*B*) PPM-2::GFP *R185A* shows increased binding to FLAG::DLK-1 *K162R* compared to wild-type PPM-2::GFP (upper panel). (*C*) Quantitation of PPM-2::GFP coIP with FLAG::DLK-1 *K162R*. Note that data was acquired from 2 independently derived transgenic lines for each genotype, and histograms represent the ratio of the amount of PPM-2::GFP or PPM-2::GFP *R185A* in coIP to the amount of FLAG::DLK-1 *K162R* that was immunoprecipitated. (*D*) Immunoblots of whole worm lysates generated solely from transgenic worms. Catalytically inactive PPM-2::GFP *D59N* was consistently expressed at elevated levels compared to wild-type PPM-2::GFP (upper panel). (*E*) Immunoblots of whole worm lysates generated solely from transgenic worms. The level of wild-type PPM-2::GFP was elevated when coexpressed with FLAG::DLK-1 *K162R*, compared to when it was coexpressed with mCherry (upper panel). (*F*) Quantitation of PPM-2::GFP levels from lysates of the indicated transgenic genotypes. Shown are the average levels of PPM-2 acquired from 4 independently derived transgenic lines for each genotype normalized to MPK-1 (loading control). Error bars represent the standard error of the mean, and significance was determined using an unpaired *t*-test. **p<0.01, ***p<0.001, ns  =  not significant.

Our genetic and transgenic experiments indicated that PPM-2 might directly dephosphorylate DLK-1. To test this hypothesis, we first determined if PPM-2 bound to DLK-1. To do so, we engineered transgenic *C. elegans* that used a pan-neuronal promoter (P*rgef-1*) to coexpress a GFP fusion protein of PPM-2 (PPM-2::GFP) with FLAG epitope tagged DLK-1 (FLAG::DLK-1). Because wild-type DLK-1 could not be expressed at sufficient levels for biochemistry (data not shown), we used a point mutant of DLK-1 *K162R* that has reduced kinase activity and can be expressed at higher levels to facilitate our analysis [80]. Both wild-type PPM-2 and catalytically inactive PPM-2 *D59N* coprecipitated with DLK-1 (Figure 5A). Thus, PPM-2 is physically associated with DLK-1 or a protein complex that contains DLK-1.

A previous study identified a conserved point mutation in mammalian PP2Cα that results in increased binding to direct targets of dephosphorylation [71]. This phosphatase trapping strategy has been used with other types of phosphatases as well [81], [82]. We observed that the corresponding point mutation in PPM-2, R185A, results in increased binding to DLK-1 *K162R* (Figure 5B). Quantitation of this interaction using multiple, independently-derived transgenic lines showed that PPM-2 *R185A* has significantly increased binding to DLK-1 (1.5+/−0.2), when compared to the level of binding between wild-type PPM-2 and DLK-1 (0.4+/−0.1, Figure 5C).

To further reinforce our model that DLK-1 is directly targeted for dephosphorylation by PPM-2, we used a combination of transgenics and biochemistry. During generation of animals with transgenic extrachromosomal arrays that used a pan-neuronal driver (P*rgef-1*) to express wild-type PPM-2::GFP or catalytically inactive PPM-2::GFP *D59N*, we noticed that the expression of PPM-2 *D59N* was elevated compared to wild-type PPM-2 (Figure 5D). This suggested that in neurons excess PPM-2 phosphatase activity is problematic, presumably due to dephosphorylation of endogenous targets. Thus, transgenic coexpression of PPM-2 with one of its direct dephosphorylation targets should result in elevated expression of PPM-2 due to titration of phosphatase activity. Consistent with this hypothesis, levels of wild-type PPM-2::GFP were increased when DLK-1 *K162R* was transgenically coexpressed, compared to when PPM-2::GFP was coexpressed with a control protein, mCherry (Figure 5E). Because whole worm lysates used for these experiments were generated exclusively from transgenic animals, it was possible to use endogenous MPK-1 (the *C. elegans* Erk1 MAPK) as a loading control. As shown in Figure 5E, the levels of MPK-1 were similar between different transgenic samples. Quantitation of PPM-2::GFP levels using multiple, independently derived transgenic lines showed that coexpression of PPM-2::GFP with FLAG::DLK-1 *K162R* resulted in a significant increase in PPM-2::GFP expression levels (2.5+/−0.5) compared to coexpression with mCherry (0.3+/−0.1, Figure 5F). Collectively, these results support the conclusion that DLK-1 is likely to be a direct target of PPM-2 phosphatase activity.

### PPM-2 acts on serine 874 in DLK-1L

**Figure 6:**
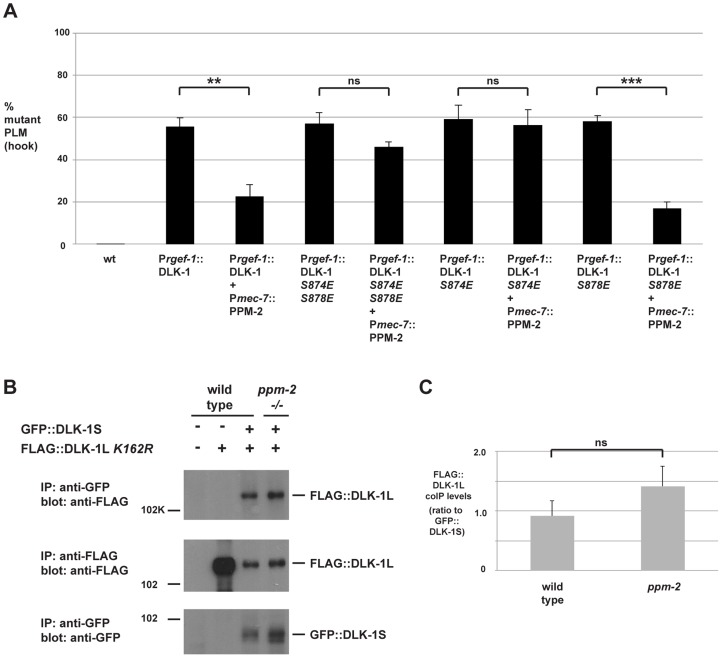
PPM-2 regulates DLK-1 by acting on S874. (*A*) Quantitation of PLM axon termination defects (hook) caused by transgenic overexpression of DLK-1 and phosphomimetic DLK-1 point mutants. Note that coexpression of PPM-2 rescues defects caused by overexpression of DLK-1, but not defects caused by overexpression of DLK-1 *S874E S878E* and DLK-1 *S874E*. Shown are averages of data pooled from 5 or more transgenic lines for the indicated genotypes; young adult worms (16–20 hours post L4) grown at 23°C were analyzed. (*B*) CoIP from transgenic whole worm lysates showing that FLAG::DLK-1L *K162R* coprecipitates with GFP::DLK-1S, and binding of DLK-1L to DLK-1S is not altered in *ppm-2-/-* mutants (upper panel). (*C*) Quantitation of FLAG::DLK-1L *K162R* coIP with GFP::DLK-1S for the indicated genotypes normalized to amount of GFP::DLK-1S precipitated. Shown are the average levels of FLAG::DLK-1 *K162R* coprecipitating with GFP::DLK-1S acquired from 3 independently derived transgenic lines for each genotype. Error bars represent the standard error of the mean, and significance was determined using an unpaired *t*-test. **p<0.01, ***p<0.001, ns  =  not significant.

A recent study showed that two conserved serine residues, S874 and S878, in the C-terminus of the long/full-length isoform of DLK-1 (DLK-1L) regulate its activity [68]. When DLK-1L is phosphorylated, it homodimerizes and is active. When DLK-1L is dephosphorylated, it preferentially binds to a short isoform of DLK-1 (called DLK-1S) creating an inactive heterodimer. Given our observation that PPM-2 is a serine/threonine phosphatase that negatively regulates DLK-1, we tested if PPM-2 functions by dephosphorylating one or both of these C-terminal serine residues in DLK-1L. To test this hypothesis, we used a transgenic approach and analyzed axon termination in the PLM neuron. We generated transgenic animals with extrachromosomal arrays that expressed either wild-type DLK-1L or a phosphomimetic point mutant of DLK-1L (*S874E S878E*) alone or in combination with PPM-2. Transgenic overexpression of DLK-1 or DLK-1 *S874E S878E* using a pan-neuronal promoter (P*rgef-1*) resulted in similar penetrance of hook defects in the PLM neurons (55.5+/−4.2% for DLK-1 and 56.8+/−5.4% for DLK-1 *S874E S878E*, Figure 6A). Coexpression of PPM-2 significantly rescued the defects caused by overexpression of DLK-1 (compare 55.5+/−4.2% for DLK-1 and 22.6+/−5.6% for DLK-1 + PPM-2), but did not rescue defects caused by overexpression of DLK-1 *S874E S878E* (compare 56.8+/−5.4% for DLK-1 *S874E S878E* and 46.1+/−2.3% for DLK-1 *S874E S878E* + PPM-2, Figure 6A). These results suggest that PPM-2 regulates phosphorylation of DLK-1L at S874, S878 or both serine residues.

We further mapped the target residue in DLK-1L that was regulated by PPM-2 by testing DLK-1L that was solely mutated at S874 or S878. As shown in Figure 6A, transgenic overexpression of DLK-1 *S874E* or DLK-1 *S878E* resulted in axon termination defects in the PLM neurons (59.1+/−6.8% for DLK-1 *S874E* and 56.1+/−7.2% for DLK-1 *S878E*). Coexpression of PPM-2 significantly rescued the defects caused by overexpression of DLK-1 *S878E* (compare 56.1+/−7.2% for DLK-1 *S878E* and 17.0+/−3.0% for DLK-1 *S878E* + PPM-2, Figure 6A). In contrast, PPM-2 did not rescue defects caused by overexpression of DLK-1 *S874E* (Figure 6A). Thus, PPM-2 regulates the activity of DLK-1L by acting on S874.

Given the results of our functional transgenic experiments, we next wanted to test if PPM-2 regulated the binding of DLK-1L to DLK-1S. We generated transgenic worms that used the pan-neuronal *rgef-1* promoter to coexpress FLAG::DLK-1L *K162R* with GFP::DLK-1S. As shown in Figure 6B, DLK-1L coprecipitates with DLK-1S, which is consistent with previous observations made using yeast two-hybrid analysis and a heterologous expression system [68]. The interaction between DLK-1L and DLK-1S was unchanged in *ppm-2-/-* mutants (Figure 6B and C).

Thus, our functional genetic and biochemical experiments demonstrate that PPM-2 acts at S874 to regulate phosphorylation and activation of DLK-1. Our results are consistent with two possible signaling models: 1) S874 is an activating phosphorylation site on DLK-1L that is regulated by PPM-2, and does not regulate binding to DLK-1S. 2) DLK-1L must be phosphorylated at both S874 and S878 to prevent binding of DLK-1S and allow DLK-1L activation.

### *ppm-2* regulates synapse formation by GABAergic motor neurons

**Figure 7:**
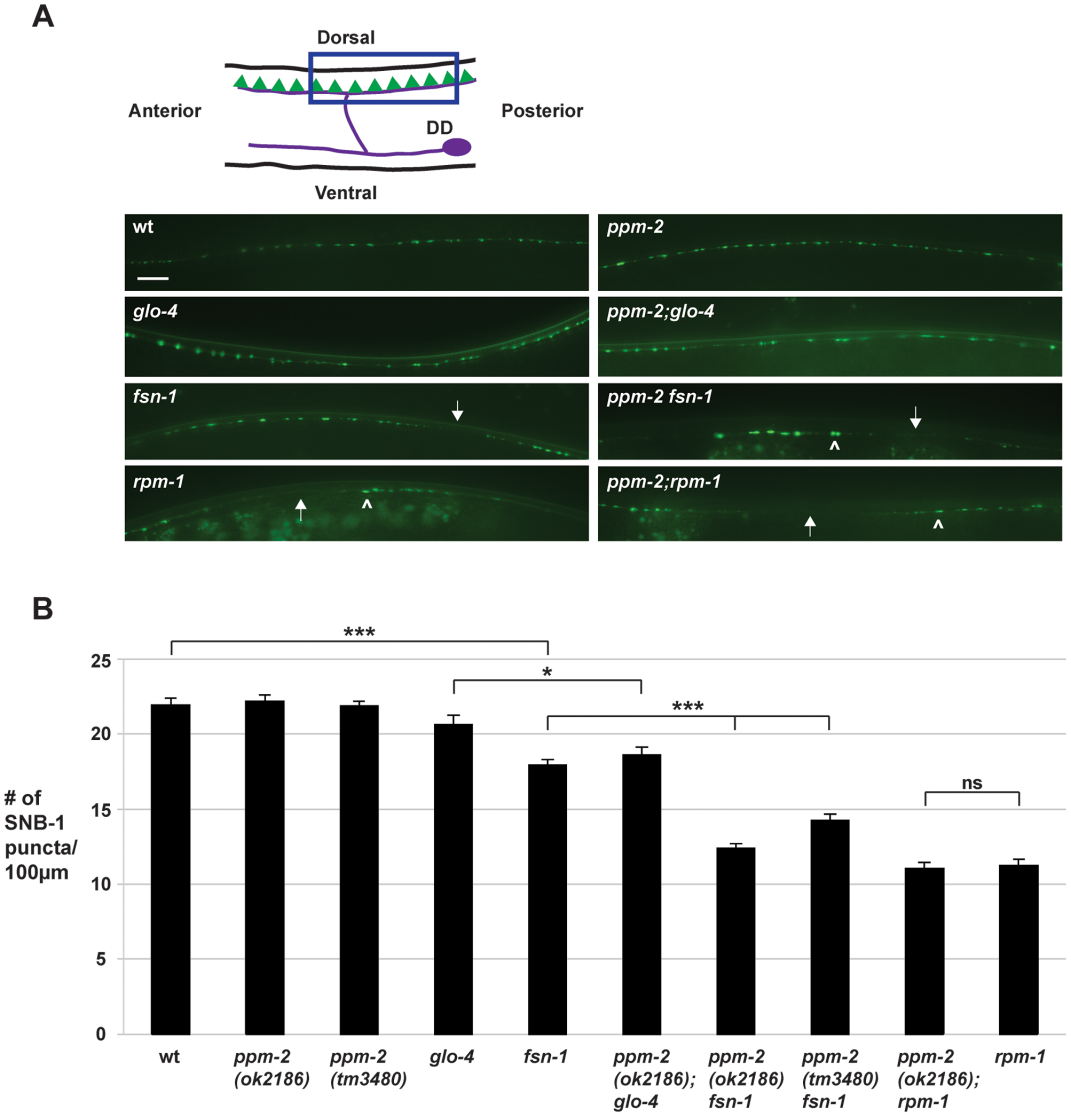
*ppm-2* regulates synapse formation by GABAergic motor neurons. (*A*) Upper panel is a schematic diagram modified from Worm Atlas showing the GABAergic DD neurons that innervate the dorsal muscles (DD cell body and axon in purple, and presynaptic terminals shown in green). A transgene, *juIs1* (P_unc-25_SNB-1::GFP), and epifluorescent microscopy was used to visualize the presynaptic terminals of the DD motor neurons for the indicated genotypes. Arrows note regions of the dorsal cord where presynaptic terminals are absent. Arrowheads highlight abnormal aggregation of presynaptic terminals. Scale bar is 10 µm. (*B*) Quantitation of synapse formation defects. Shown are averages for data collected from 3 or more independent experiments performed at 25°C in which 15–20 synchronized, young adult worms (16–20 hours post L4) were analyzed. Error bars represent the standard error of the mean, and significance was determined using an unpaired *t*-test. *p<0.05, ***p<0.001 and ns  =  not significant.

Previous studies in *C. elegans* have shown that *rpm-1* regulates synapse formation in motor neurons [31], [38]. In *C. elegans*, there are two sets of GABAergic, inhibitory motor neurons: the VD neurons that innervate the ventral muscles, and the DD neurons that innervate the dorsal muscles (Figure 7A, see schematic). The presynaptic terminals of VD and DD motor neurons were visualized in living worms using *juIs1*, a transgene that uses a cell-specific promoter (P*unc-25*) to drive expression of a fusion protein of Synaptobrevin 1 (SNB-1) and GFP (SNB-1::GFP) [83]. In wild-type animals, SNB-1::GFP localized to presynaptic puncta that were uniform in size and evenly spaced along the dorsal nerve cord (Figure 7A). In *rpm-1-/-* mutants, the dorsal SNB-1::GFP puncta were abnormal in size and aggregated (Figure 7A, arrowheads), and there were sections of the dorsal cord with gaps in which no SNB-1::GFP puncta were present (Figure 7A, arrows). Previous studies using electron microscopy established that defects in the localization of SNB-1::GFP in *rpm-1-/-* mutants reflect defects in synapse formation, rather than simply defects in the formation of presynaptic terminals or the trafficking of synaptic vesicles [31], [38]. Quantitation of the number of SNB-1::GFP puncta showed that *rpm-1-/-* animals had fewer synapses than wild-type animals (compare 11.3+/−0.4 SNB-1::GFP puncta/100 µm for *rpm-1* to 21.9+/−0.4 puncta/100 µm for wild type, Figure 7B). This observation was consistent with previous studies [38], [75].

To test if *ppm-2* functions in synapse formation, we analyzed *ppm-2-/-* animals and a series of double mutants of *ppm-2* and members of the RPM-1 pathway. We found that *ppm-2(ok2186)-/-* and *ppm-2(tm3480)-/-* mutants had normal patterning and numbers of SNB-1::GFP puncta (Figure 7A and B). In contrast, *ppm-2-/- fsn-1-/-* and *ppm-2-/-; glo-4-/-* double mutants had enhanced defects in synapse formation (Figure 7A) compared to single mutants (compare 14.2+/−0.5 puncta/100 µm for *ppm-2(tm3480) fsn-1* and 12.4+/−1.7 for *ppm-2(ok2186) fsn-1* to 17.9+/−0.4 for *fsn-1*, Figure 7B). We also analyzed *ppm-2-/-; rpm-1-/-* double mutants and found that they had similar defects in synapse formation to *rpm-1-/-* single mutants assessed qualitatively (Figure 7A) and quantitatively (Figure 7B). Our observations are consistent with two conclusions. First, *ppm-2* functions in a parallel genetic pathway to both *fsn-1* and *glo-4* to regulate synapse formation, since all the mutants used in our study are null alleles. Second, *ppm-2* functions in the same genetic pathway as *rpm-1* to regulate synapse formation, as synapse formation defects in *ppm-2-/-; rpm-1-/-* double mutants were not enhanced, and the synapse formation defects in *rpm-1*-/- mutants are not saturated [51].

### PPM-2 is localized to presynaptic terminals

**Figure 8:**
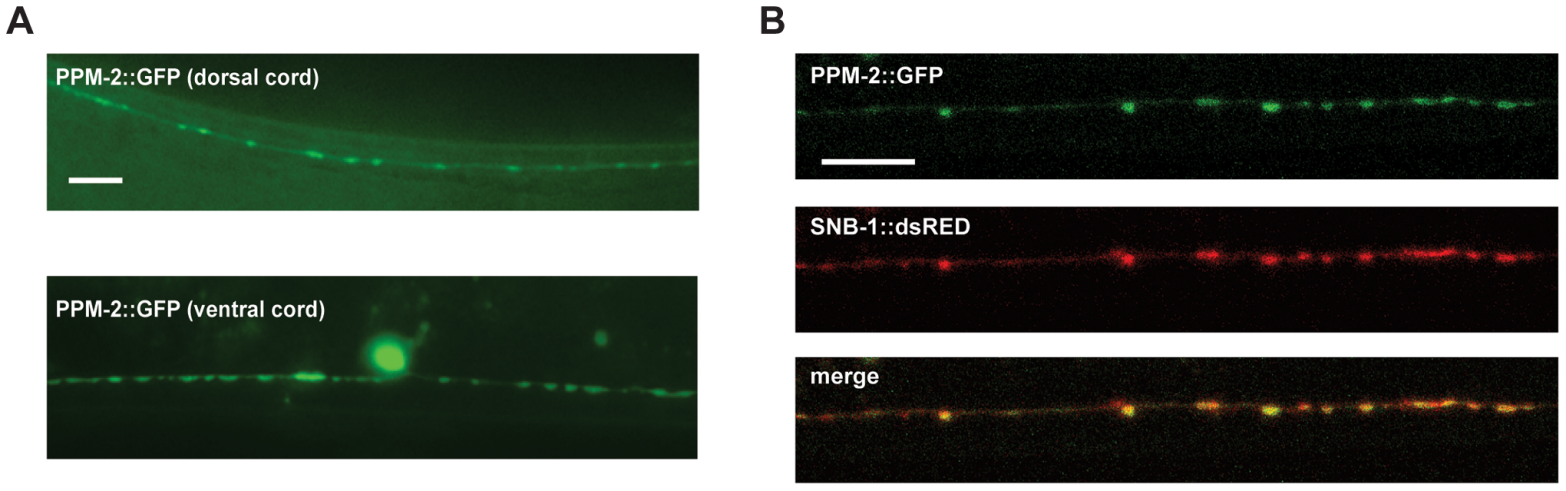
PPM-2 localizes to the presynaptic terminal. (*A*) PPM-2::GFP was transgenically expressed in the GABAergic motor neurons using a cell specific promoter (P*unc-25*). Epifluorescent microscopy was used to visualize PPM-2::GFP puncta in the dorsal and ventral cords. (*B*) Transgenic worms expressing PPM-2::GFP and SNB-1::dsRED in the GABAergic motor neurons were analyzed by confocal microscopy. Shown are the presynaptic terminals of the DD neurons on the dorsal side of the animal. Scale bar is 10 µm.

Having established the molecular mechanism of how PPM-2 functions in neuronal development, we wanted to determine if PPM-2 is localized to a particular subcellular compartment in neurons. To do so, we took a transgenic approach in which PPM-2 was expressed in the GABAergic motor neurons. The GABAergic motor neurons were analyzed to maintain consistency with prior cell biology studies on RPM-1 and its binding partners. A cell specific promoter (P*unc-25*) was used to express PPM-2 as a C-terminal fusion protein with GFP (PPM-2::GFP), in order to ensure that GFP did not interfere with the myristoylation of PPM-2. PPM-2::GFP was localized broadly throughout the motor neuron axons and cell bodies with strong enrichment in puncta along the ventral and the dorsal nerve cord (Figure 8A). This localization pattern was similar to molecules that are localized to the presynaptic terminal, such as SNB-1. To test this possibility, transgenic animals were generated that expressed a fusion protein of SNB-1 and dsRED (SNB-1::dsRED) with PPM-2::GFP in the GABAergic motor neurons. As shown in Figure 8B, PPM-2::GFP and SNB-1::dsRED colocalized in puncta at the presynaptic terminals of the DD neurons on the dorsal side of the animal. Localization of PPM-2 to the presynaptic terminal is consistent with our finding that PPM-2 regulates synapse formation in the GABAergic motor neurons.

## Discussion

How axon outgrowth, synapse formation, and termination of axon outgrowth are molecularly coordinated during development remains poorly understood. The PHR proteins meet a growing list of criteria as molecules that integrate and coordinate these events during development. The PHR proteins function in synapse formation [24], [29], [31], [32], axon guidance and outgrowth [20], [22], [25], and axon termination [22], [29], [30], and importantly often do so in the same type of neuron. Studies using *C. elegans* and Drosophila have shown that PHR proteins are extremely large, intracellular signaling proteins that function cell autonomously. Thus, PHR proteins have the potential to integrate signals coming from different extracellular cues converging on a single neuron. There is evidence suggesting that the activity of the PHR proteins can be regulated [43], [44], although the extracellular signals that activate or inhibit PHR proteins remain unclear. Finally, the PHR proteins negatively and positively regulate multiple downstream signaling pathways that control gene transcription [22], [38], [41], [46], [84], signal transduction and local mRNA translation [39], [40], [42], [46], [50], microtubule dynamics [22], [26], [45], and vesicle trafficking/formation [39].

**Figure 9:**
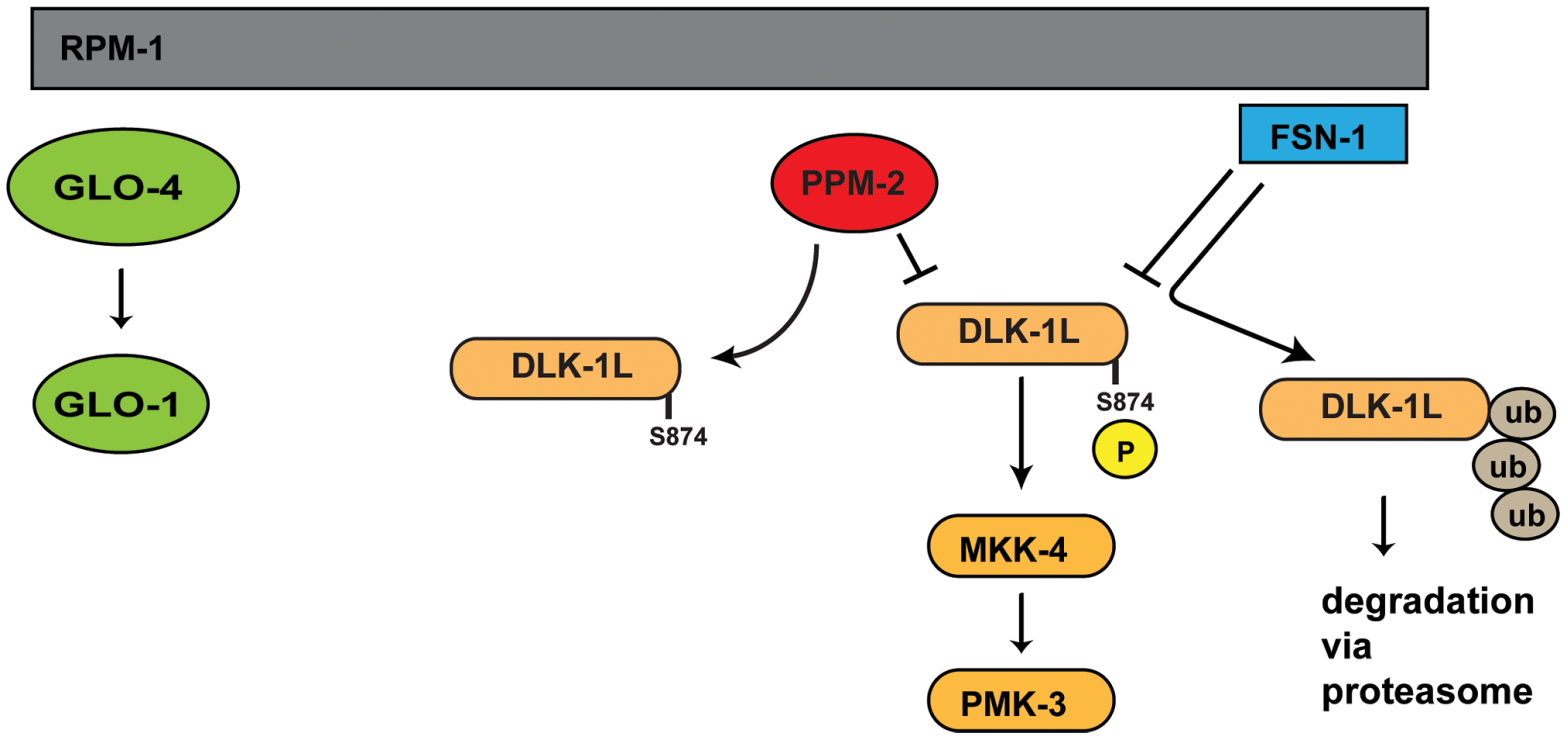
Summary of RPM-1 signaling. RPM-1 is a positive regulator of the GLO-4 pathway, and acts as part of a complex with FSN-1 that ubiquitinates and negatively regulates DLK-1. PPM-2 is also part of an RPM-1 protein complex and negatively regulates DLK-1 via dephosphorylation at S874.

Studies in worms, flies, fish, and mice have shown that PHR proteins function as ubiquitin ligases to negatively regulate the MAP3K DLK, which represents a mechanism to control long-term signaling by the DLK pathway. We now show that *C. elegans* RPM-1 also utilizes a phosphatase, PPM-2, to negatively regulate DLK-1 (Figure 9). This suggests that RPM-1 has the potential to rapidly regulate signaling by DLK-1. Thus, our results suggest that RPM-1 has the potential to employ different regulatory mechanisms to spatially and/or temporally regulate DLK-1 signaling, which provides further support for the proposition that PHR proteins may coordinate different events during development. To our knowledge, RPM-1 also now represents the first example of a single signaling protein that controls both ubiquitination and dephosphorylation of a MAP3K.

### PPM-2 mediates RPM-1 function

Our proteomic screen for RPM-1 binding proteins identified the PP2C phosphatase PPM-2, which we confirmed using coIP from transgenic *C. elegans*. Genetic and transgenic experiments showed that *ppm-2* functions downstream of *rpm-1* to regulate axon termination and synapse formation. Importantly, our genetic analysis also demonstrated that *ppm-2* and *rpm-1* function in the same genetic pathway consistent with our observation that these molecules physically interact.

Axon termination and synapse formation defects were enhanced in *ppm-2-/- fsn-1-/-* double mutants and *ppm-2-/-;glo-4-/-* double mutants. Given that we analyzed null alleles of *ppm-2*, *glo-4*, and *fsn-1*, our results are consistent with these three molecules acting in parallel pathways that converge on a common molecular player or process in neurons. In the case of PPM-2 and FSN-1, we have identified this common player as DLK-1. It remains unclear which downstream molecule or process is commonly regulated by GLO-4 and PPM-2.

Our results support the conclusion that RPM-1 positively regulates PPM-2 for several reasons. First, *rpm-1* and *ppm-2* (lf) mutants both have axon termination defects. Second, defects in *ppm-2-/-; rpm-1-/-* double mutants were not suppressed. If PPM-2 were negatively regulated by RPM-1, we would expect that loss of function in *ppm-2* would suppress the defects in *rpm-1* mutants. Such is the case for DLK-1, which is negatively regulated by RPM-1 [38]. Finally, none of the molecules we have identified in our proteomic screen for RPM-1 binding proteins (including: GLO-4, FSN-1, and RAE-1) have been genetic suppressors of *rpm-1* (lf) [39], [45]. Thus, our screen does not efficiently identify ubiquitination targets of RPM-1, presumably because such interactions are transient. Overall, our data are consistent with the conclusion that RPM-1 binds to and positively regulates PPM-2. Nonetheless, developing a more refined mechanistic understanding of how PPM-2 is regulated by RPM-1 remains an important goal.

RPM-1 is highly conserved with functional orthologs in Drosophila, zebrafish, and mice. The RPM-1 binding proteins we previously identified in our proteomic screen, including GLO-4, FSN-1 and RAE-1, are also evolutionarily conserved from worms to mammals [39], [45]. With regard to PPM-2, we identified clear orthologs in Drosophila and the protochordate *Ciona intestinalis*, which suggests that PPM-2 is likely to represent a conserved mechanism by which PHR proteins function. However, due to lack of sequence conservation we were unable to identify an orthologous phosphatase to PPM-2 in vertebrates. While PPM-2 may represent a unique mechanism of regulating neuronal development in invertebrates and protochordates, it is also possible that another PP2C phosphatase (or a member of a different phosphatase family) may perform the function of PPM-2 in vertebrate neurons. It is notable that the PP2C phosphatase PPM-1, which is one of the closest homologs of PPM-2 in *C. elegans*, also regulates axon termination and synapse formation [75]. In mammals, PPM-1 has three orthologs, PP2Cα, PP2Cβ, and PP2Cβ-like. Because the PPM-1 subfamily has undergone significant expansion in mammals, PP2Cα, PP2Cβ, and PP2Cβ-like are plausible candidates as the functional ortholog of PPM-2. Future experiments in *C. elegans* using mammalian PP2C phosphatases may be helpful in addressing this possibility.

### A complex negative regulatory network controls the DLK-1 pathway

RPM-1 and the PHR proteins function in part by ubiquitinating and negatively regulating the MAP3K DLK-1 [22], [38], [41]. Transgenic overexpression of DLK-1 at higher levels than used in our study causes more severe defects than *rpm-1* (lf), such as uncoordinated locomotion and small body size [38], [80]. This observation suggested that negative regulatory mechanisms, aside from ubiquitination by RPM-1, restrain the activity of DLK-1 and/or its downstream kinases. Recent work in *C. elegans* has supported this hypothesis by showing that PPM-1 (a homolog of PPM-2) and VHP-1 (a dual specificity phosphatase) negatively regulate the DLK-1 pathway [75], [85]. Nonetheless, it remained uncertain if a specific phosphatase directly regulated DLK-1. It also remained unclear if RPM-1 functions through mechanisms other than ubiquitination to control the activity of DLK-1.

We now address both these questions by showing that RPM-1 binds to the phosphatase PPM-2, and functions through this phosphatase-based mechanism to negatively regulate DLK-1 (Figure 9). Our biochemical and functional genetic analysis indicate that DLK-1 is negatively regulated by PPM-2. This is most likely the result of direct dephosphorylation as corroborated by several observations: 1) PPM-2 binds to DLK-1. 2) PPM-2 *R185A* acts as a trap for phosphorylation targets, and shows increased binding to DLK-1. 3) Transgenic expression of DLK-1 allows increased coexpression of PPM-2, presumably by titrating excess PPM-2 phosphatase activity that is otherwise problematic for neurons.

A previous study showed that phosphomimetic point mutation of DLK-1L at both S874 and S878 allowed DLK-1L to homodimerize and become active [68]. Point mutations in DLK-1L that prevented phosphorylation at both S874 and S878 resulted in formation of an inactive heterodimer with DLK-1S (short, inhibitory isoform). This prior study did not determine if phosphorylation of DLK-1L at S874, S878 or both residues was required to regulate binding to DLK-1S. We have found that PPM-2 acts specifically on S874 to regulate DLK-1L activity (Figure 9). While PPM-2 activity is sufficient to inhibit transgenically overexpressed DLK-1 (Figure 6A), it was not sufficient to regulate binding of DLK-1L to DLK-1S (Figure 6B). This result is consistent with two possible interpretations. 1) Phosphorylation of DLK-1 at S874 is required for activation of DLK-1, but this activation occurs through a mechanism that is independent of DLK-1S. 2) DLK-1L needs to be phosphorylated at both S874 and S878 to block binding to DLK-1S and become active. In this case, DLK-1L would display different levels of inactivation, with dephosphorylation at both residues presumably being the most inactive. Such a signaling model would allow sophisticated spatial and temporal control over how quickly DLK-1 is activated. A combination of future biochemical and genetic experiments will hopefully support one of these two signaling models.

*C. elegans* DLK-1 has two homologs in mammals, DLK (MAP3K12) and LZK (MAP3K13) both of which are highly expressed in brain [86], [87]. While DLK plays an important role in neuronal development and axon regeneration, our knowledge regarding LZK remains relatively modest. Notably, the small segment of the *C. elegans* DLK-1L C-terminus that contains S874 and S878 is conserved with mammalian LZK, and not mammalian DLK [68]. Thus, the mammalian functional ortholog of PPM-2 would be likely to regulate LZK rather than DLK.

Because RPM-1 binds to and positively regulates PPM-2, it is plausible that RPM-1 acts as a more sophisticated regulator of DLK-1 than originally thought by regulating long-term DLK-1 signaling (through ubiquitination) and short-term/local DLK-1 signaling (through the phosphatase activity of PPM-2). The idea that RPM-1 controls local/short-term signaling and long-term signaling is supported by observations in flies and worms showing that the DLK-1 pathway regulates the activity of the transcription factors Fos and CEBP-1 [40], [41], and the translation of CEBP-1 locally in axons [40]. We note that our results cannot absolutely rule out the possibility that dephosphorylation of DLK-1 by PPM-2 is a prerequisite for ubiquitination and degradation of DLK-1 by an RPM-1/FSN-1 ligase complex. However, our finding that *ppm-2* and *fsn-1* function in parallel genetic pathways makes this extremely unlikely.

It remains important to address how RPM-1 determines whether to degrade DLK-1, or act through PPM-2 to dephosphorylate DLK-1. This may be based on developmental timing, the subcellular location of DLK-1, the activation state of DLK-1, post-translational modification of DLK-1, or a combination of these factors. Upstream signals may also instruct RPM-1 to degrade or dephosphorylate DLK-1. A better understanding of the mechanisms and molecules that activate RPM-1 and DLK-1 are likely to be helpful in addressing these possibilities.

Since both PPM-2 and PPM-1 negatively regulate the DLK-1 pathway, we attempted to analyze the genetic relationship between *ppm-2* and *ppm-1*. Unfortunately, *ppm-2-/-; ppm-1-/-* double mutants were embryonic lethal in the F2 generation (data not shown), which rendered our genetic analysis inconclusive. However, the synthetic lethality observed between *ppm-2* (lf) and *ppm-1* (lf) is consistent with these phosphatases acting on different targets. This agrees with our prior study that suggested PPM-1 acts at the level of the p38 MAP kinase PMK-3 [75], and our work here demonstrating that PPM-2 acts on DLK-1. Several other pieces of evidence support the conclusion that PPM-2 and PPM-1 act on different targets in the DLK-1 pathway. The primary phenotype in *ppm-2-/-* animals was low penetrance hook defects in PLM neurons, while we previously showed that *ppm-1-/-* mutants primarily display low penetrance overextension defects in PLM neurons [75]. Since the hook phenotype is more severe [39], [45], our results are consistent with PPM-2 acting higher up in the kinase cascade than PPM-1. This model is further supported by our observation that *ppm-2-/- fsn-1-/-* double mutants show stronger enhancement of hook defects (Figure 2D) compared to *ppm-1-/-; fsn-1-/-* double mutants [75]. Nonetheless, further biochemical and transgenic studies will be needed to definitively determine if PPM-2 and PPM-1 act at different points in the DLK-1 pathway.

### N-myristoylation and PPM-2

Previous studies have shown that several types of phosphatases are myristoylated [88]–[91]. Our mass spectrometry and transgenic results indicated that N-myristoylation is also required for PPM-2 to be fully functional. Our findings are consistent with prior observations on PP2C phosphatases. First, other PP2C phosphatases have N-myristoylation consensus motifs [92]. Second, a recent biochemical study showed that two PP2C phosphatases (PPM1A and PPM1B) are N-myristoylated, and myristoylation is required for substrate specificity, but not enzymatic activity [91]. However, myristoylation is also known to regulate the membrane localization of signaling proteins. Thus, our findings coupled with these prior observations demonstrate that N-myristoylation is an important posttranslational modification that mediates the substrate specificity and/or membrane localization of PPM-2. We also show for the first time that myristoylation is important for the function of PP2C phosphatases in neurons.

Previous work showed that mammalian DLK is associated with the plasma membrane at synapses [63]. While DLK in the cytosol is in both hyperphosphorylated and unphosphorylated forms, membrane bound DLK is phosphorylated to a lesser extent or unphosphorylated. Our observations that PPM-2 is N-myristoylated, that PPM-2 negatively regulates DLK-1, and that PPM-2 binds to DLK-1 suggest that PPM-2 may act at the plasma membrane of the presynaptic terminal to regulate the phosphorylation of DLK-1. This model is consistent with our observation that PPM-2 is localized to the presynaptic terminal (see Figure 8). Alternatively, N-myristoylation may regulate the target specificity of PPM-2 and, therefore, PPM-2 activity on DLK-1.

### PPM-2 function outside of neuronal development

While DLK-1 plays an important role in neuronal development, it is also essential for axon regeneration in *C. elegans*, Drosophila, and mice [33], [34], [40], [58], [59]. Importantly, loss of function in *C. elegans rpm-1* or Drosophila *Highwire*, or overexpression of DLK-1, lead to improved axon regeneration [33], [34], [40], [85]. Loss of function in *fsn-1* also results in improved regeneration [33]. These studies demonstrate that relieving inhibition on or activating the DLK-1 pathway promotes axon regeneration, particularly in older adult *C. elegans* where regeneration is less robust.

Our finding that PPM-2 negatively regulates DLK-1 during neuronal development suggests that PPM-2 may also play a post-developmental role in axon regeneration. Our findings predict that increased DLK-1 activity in *ppm-2-/-* animals may result in improved regenerative capacity compared to wild-type animals. In addressing this possibility, two important considerations from our developmental understanding of DLK-1 signaling should be taken into account. 1) A combination of loss of function in *fsn-1* and *ppm-2* may be necessary to see improved regeneration, as these two genes function in parallel pathways to regulate DLK-1. 2) A prior study showed that older animals have poor regeneration that is more sensitive to increased levels of DLK-1 activity [33]. Therefore because PPM-2 is a partial regulator of DLK-1 activity, the possible effects of PPM-2 on regeneration may be more readily detected in older animals. Exploring the possible role of PPM-2 (and other PP2C phosphatases) in regeneration is worthwhile, as the enzymatic activity of PP2C phosphatases may be particularly amenable to pharmaceutical intervention.

Recent work in *C. elegans* has shown that axon regeneration is regulated by another MAPK pathway composed of MLK-1, MEK-1, and the JNK kinase KGB-1 [85]. While the role of the MLK-1 pathway in neuronal development remains unexplored, it is possible that PP2C phosphatases, such as PPM-2 and PPM-1, may also regulate this pathway.

### Conclusion

We now provide evidence that RPM-1 is potentially a more sophisticated regulator of DLK-1 than originally thought. While this is a significant step forward, important questions remain: 1) Does RPM-1 regulate molecules other than DLK-1 in a more precise and accurate manner, possibly through PPM-2 or other mechanisms? 2) How is RPM-1 activity regulated, and do upstream extracellular guidance cues, morphogens, or adhesion molecules instruct the activity of RPM-1 during neuronal development? 3) Is RPM-1 located in multiple subcellular compartments in a single neuron, which may explain the dual role of RPM-1 in axon termination and synapse formation? Addressing these questions will be essential to further support the previously proposed model that RPM-1 and the PHR proteins function to coordinate different events in neuronal development [20], [21].

## Materials and Methods

### Genetics

The N2 isolate of C. elegans was propagated using standard procedures. The alleles used in this study included: rpm-1(ju44), fsn-1(gk429), glo-4(ok623), dlk-1(ju476), ppm-2(ok2186), ppm-2(tm3480), unc-32(e189), unc-103(e1597), dpy-17(e164), and lon-1(e1820). For generation of transheterozygous ppm-2(ok2186)/ppm-2(tm3480) fsn-1 double mutants, tm3480 was linked to unc-32(e189) and ok2186 was linked to lon-1(e1820). Transheterozygous animals were identified as non-lon, non-unc animals. ppm-2(ok2186) fsn-1 and ppm-2(tm3480) fsn-1 double mutants were constructed by recombination using ok2186 and tm3480 linked to dpy-17(e164) and fsn-1 linked to unc-103(e1597).

### Transgenics

Transgenic animals were generated by standard microinjection procedures. All transgenes were constructed by injection of plasmid or DNA generated by PCR with P_ttx-3_RFP (50 ng/µL) or P_myo-2_mCherry (2.5 ng/µL) and pBluescript (50 ng/µL). Injection conditions and genotypes for all transgenes are annotated in Table S1. For all transgenic and biochemical experiments, a cDNA encoding isoform B of DLK-1 was used. Point mutants of DLK-1 (S874E and S878E) were annotated based on isoform A for continuity of presentation with previous studies, but these residues correspond to S867 and S871 in isoform B.

### Biochemistry and mass spectrometry

Purification of RPM-1::GFP from transgenic *C. elegans* and identification of associated RPM-1 binding proteins by mass spectrometry was described previously [39]. In brief, transgenic mixed stage *C. elegans* were grown in liquid cultures using M9 buffer supplemented with cholesterol and HB101 *E. coli*. Worms were washed repeatedly in fresh M9 buffer, and pelleted by low speed centrifugation. Frozen worms were ground under liquid N_2_ with a mortar and pestle, and extracted with 0.1% NP-40 lysis buffer (10 mM Tris pH 7.4, 0.1% NP-40, 150 mM NaCl, 1 mM DTT, protease and phosphatase inhibitors). GFP::RPM-1 was immunoprecipitated from worm extracts using an anti-GFP antibody (3E6 mouse monoclonal, Qbiogene), run on an SDS-PAGE gel, and proteins coprecipitating with GFP::RPM-1 were identified by LC-MS/MS.

For coIP of RPM-1 and PPM-2, worms were lysed in 0.1% NP-40 lysis buffer (50 mM Tris pH 7.5, 0.1% NP-40, 150 mM NaCl, 10% glycerol, protease inhibitors, and phosphatase inhibitors: microcystin, NaVO4, NaF, NaMolybdate, and β-glycerophosphate). For coIP, transgenic proteins were immunoprecipitated with an anti-FLAG antibody (M2 mouse monoclonal, Sigma) or an anti-GFP antibody (3E6 mouse monoclonal, MP Biomedicals) and protein G agarose. Coprecipitating GFP fusion proteins or FLAG tagged proteins were detected by immunoblotting with an anti-GFP antibody (Roche, mouse monoclonal) or an anti-FLAG antibody (rabbit monoclonal, Cell Signaling), respectively. 3 mg of total worm lysate was used for coIP of FLAG::PPM-2 with RPM-1::GFP. 5 mg of total worm lysate was used for coIP of wild-type or mutant PPM-2::GFP with FLAG::DLK-1 *K162R*. 10 mg of total worm lysate was used for coIP of GFP::DLK-1S with FLAG:DLK-1L *K162R*.

For immunoblot analysis of whole *C. elegans* lysates, samples were prepared as described previously [93]. Animals were age-synchronized by bleach treatment. Array positive young adults were selected using P_ttx-3_RFP and cleaned by transferring to an NGM plate with no bacteria. 50 animals were picked for each genotype and placed into a tube with 20 µL of water. Samples were mixed with 20 µL of 2X Laemmli Sample Buffer (Biorad) with 2-βmercaptoethanol. Animals were pelleted by centrifugation and samples were flash frozen in liquid nitrogen. Prior to immunoblotting, samples were incubated at 95°C for 5 minutes, cooled for 5 minutes at room temperature, and loaded onto an SDS-PAGE gel. GFP fusion or FLAG tagged proteins were detected by immunoblotting with an anti-GFP antibody (Roche, mouse monoclonal) or an anti-FLAG antibody (rabbit monoclonal, Cell Signaling), respectively. MPK-1 (Erk1) was detected by immunoblotting with an anti-ERK1 antibody (K-23 rabbit polyclonal, Santa Cruz Biotechnology) and mCherry was detected by immunoblotting with an anti-mCherry antibody (mouse monoclonal, Clontech).

Immunoblots from coIP and whole cell lysates were visualized using secondary antibodies coupled to horseradish peroxidase, Supersignal FemtoWest chemiluminescent reagent (Pierce), and x-ray film. Western Lightning *Plus*-ECL (PerkinElmer) was used for anti-Erk immunoblotting of MPK-1. Quantitation of bands in immunoblots of whole worm lysates and coIP experiments was performed using image J software from NIH image (http://rsb.info.nih.gov/ij/).

### Axon termination and synapse formation analysis

For analysis of GFP or SNB-1::GFP, live young adult animals (16–20 hours post L4) were anesthetized using 1% (v/v) 1-phenoxy-2-propanol in M9 buffer and visualized using a 40x magnification oil-immersion lens and an epifluorescent microscope (Nikon Eclipse E40). Images were acquired using a CCD camera (Q-imaging Qicam 1394). Axon termination defects in the mechanosensory neurons were quantified by manually scoring *muIs32* (P_mec-7_GFP). Synapse formation defects were quantified by collecting images of *juIs1* (P_unc-25_SNB-1::GFP), and manually scoring puncta numbers in acquired images using Adobe Photoshop. The mechanosensory neurons were analyzed at 23°C, as previous studies showed that axon termination defects in PLM neurons are maximally defective at 23°C [29], [39]. While ALM axon termination and PLM synaptic branch extension are temperature sensitive processes, 23°C is a sufficient temperature to achieve strong phenotypes. Synapse formation in GABAergic neurons was analyzed at 25°C because a previous study showed that the *ju44* allele of *rpm-1* is temperature sensitive with regard to this process, and gives a maximal phenotype at 25°C [31].

### Confocal microscopy

Colocalization of SNB-1::dsRED and PPM-2::GFP (*bggEx88*) was analyzed using a Zeiss 780 laser scanning confocal microscope at 63x magnification under immersion oil. Images were acquired using Zeiss Zen software, and analyzed using Image J. Young adult animals were analyzed in all cases.

## Supporting Information

Figure S1 PPM-2 peptides identified by proteomics. Our proteomic screen for RPM-1 binding proteins identified 5 unique peptides for PPM-2 (highlighted in red/blue) that covered 15% of the total PPM-2 protein sequence.
(TIF)

Figure S2 *ppm-2* regulates synaptogenesis, as assessed by synaptic branch extension, in the PLM mechanosensory neurons. The synaptic branch of the PLM neurons was visualized using the transgene *muIs32* (P_mec-7_GFP). (*A*) Epifluorescent microscopy was used to visualize the synaptic branch in wild-type or *rpm-1-/-* mutant animals. The images shown correspond to the boxed region of the diagram. Note the absence of the synaptic branch in *rpm-1-/-* mutants (arrow). Scale bar is 10 µm. (*B*) Quantitation of the defects in synaptic branch extension in the PLM neurons for the indicated genotypes. Averages are shown for data collected from 5–8 independent counts of 20–30 PLM neurons from adult worms grown at 23°C. Error bars represent the standard error of the mean, and significance was determined using an unpaired t-test. *** p<0.001 and ns  =  not significant.
(TIF)

Figure S3 *ppm-2* regulates axon termination in the ALM mechanosensory neurons. The axons of ALM mechanosensory neurons were visualized using the transgene *muIs32* (P_mec-7_GFP). (*A*) Epifluorescent microscopy was used to visualize the ALM axon in wild-type or *rpm-1-/-* mutants. The images shown correspond to the boxed region of the diagram. Note that in *rpm-1-/-* mutants two types of axon termination defects are visible: 1) more severe big hooks in which the axon overextends and hooks to the posterior of animal (top panel), and 2) less severe short hooks in which the axon overextends more modestly, and does not extend towards the posterior (lower panel). (*B*) Quantitation of specific, short hook (gray) or big hook (black), axon termination defects in the ALM mechanosensory neurons of the indicated genotypes. Averages are shown for data collected from 5–8 independent counts of 20–30 ALM neurons from adult worms grown at 23°C. Error bars represent the standard error of the mean, and significance was determined using an unpaired t-test. ***p<0.001 and ns  =  not significant.
(TIF)

Figure S4 N-myristoylation of PPM-2. (*A*) Shown is a mass spectrum of the PPM-2 N-terminus peptide. Note that the size of ions b1, b2, b3 and b4 are shifted in size by 210 Da indicating that this PPM-2 peptide is N-myristoylated. (*B*) PPM-2 shares a conserved N-myristoylation site (G2) with several other indicated PP2C family phosphatases. Sequence analysis was done using EMBL-EBI ClustalW2. * (asterisk) indicates positions that are identical, (colon) indicates residues that show conservation between strongly similar amino acids, and. (period) indicates conservation between weakly similar amino acids.
(TIF)

Table S1 A list of transgenic *C. elegans* strains used in this study. * Alleles used are *ppm-2(ok2186), fsn-1 (gk429), dlk-1 (ju476)*, and *rpm-1(ju44)*. ∧ Strains were constructed by injection of plasmid DNA, unless noted.
(DOCX)
