# Supplementary figures and images for: RPM-1 Uses Both Ubiquitin Ligase and Phosphatase-Based Mechanisms to Regulate DLK-1 during Neuronal Development
Source: PLoS Genet. 2014 May 8;10(5):e1004297. doi: 10.1371/journal.pgen.1004297 (PMC4014440; doi:10.1371/journal.pgen.1004297)

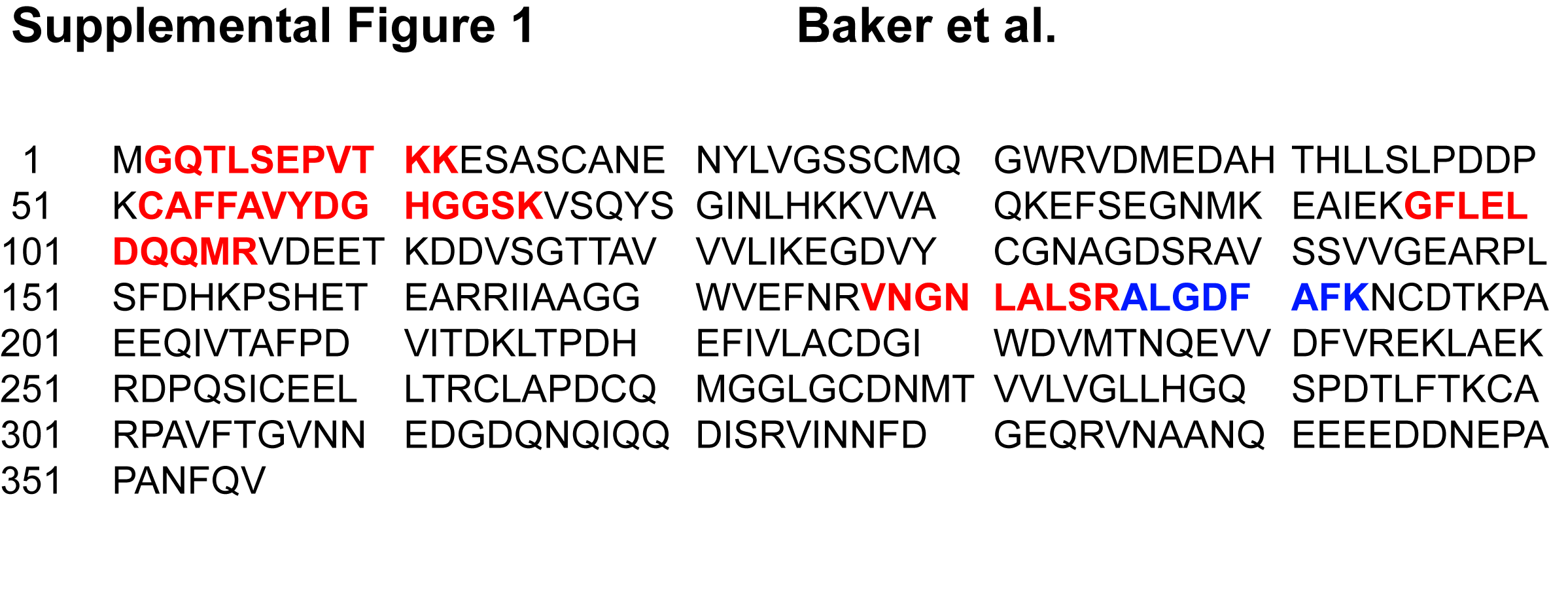

Supplement: Figure S1 — PPM-2 peptides identified by proteomics. Our proteomic screen for RPM-1 binding proteins identified 5 unique peptides for PPM-2 (highlighted in red/blue) that covered 15% of the total PPM-2 protein sequence. (TIF) [file pgen.1004297.s001.tif]

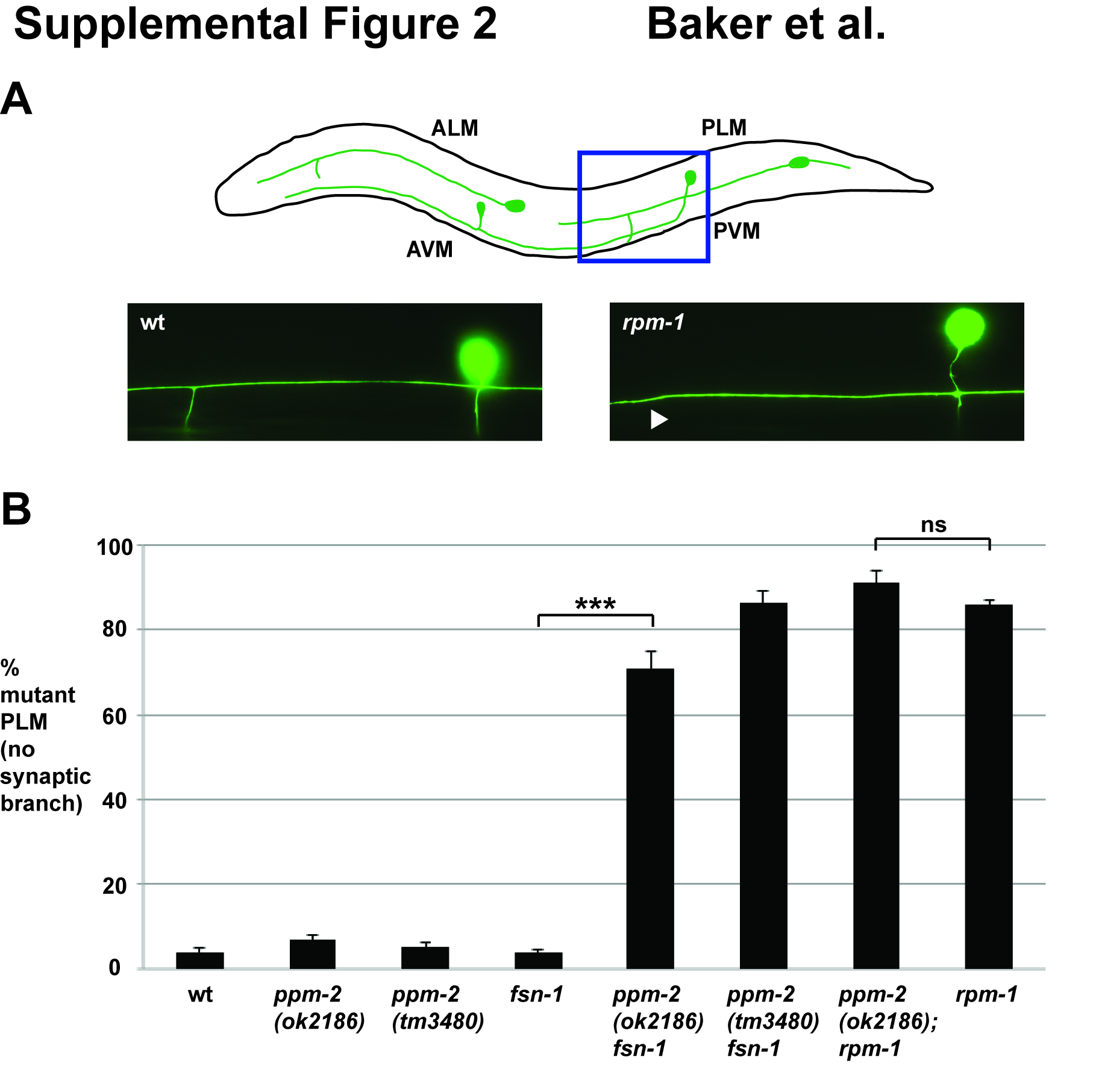

Supplement: Figure S2 — ppm-2 regulates synaptogenesis, as assessed by synaptic branch extension, in the PLM mechanosensory neurons. The synaptic branch of the PLM neurons was visualized using the transgene muIs32 (Pmec-7GFP). (A) Epifluorescent microscopy was used to visualize the synaptic branch in wild-type or rpm-1-/- mutant animals. The images shown correspond to the boxed region of the diagram. Note the absence of the synaptic branch in rpm-1-/- mutants (arrow). Scale bar is 10 µm. (B) Quantitation of the defects in synaptic branch extension in the PLM neurons for the indicated genotypes. Averages are shown for data collected from 5–8 independent counts of 20–30 PLM neurons from adult worms grown at 23°C. Error bars represent the standard error of the mean, and significance was determined using an unpaired t-test. *** p<0.001 and ns = not significant. (TIF) [file pgen.1004297.s002.tif]

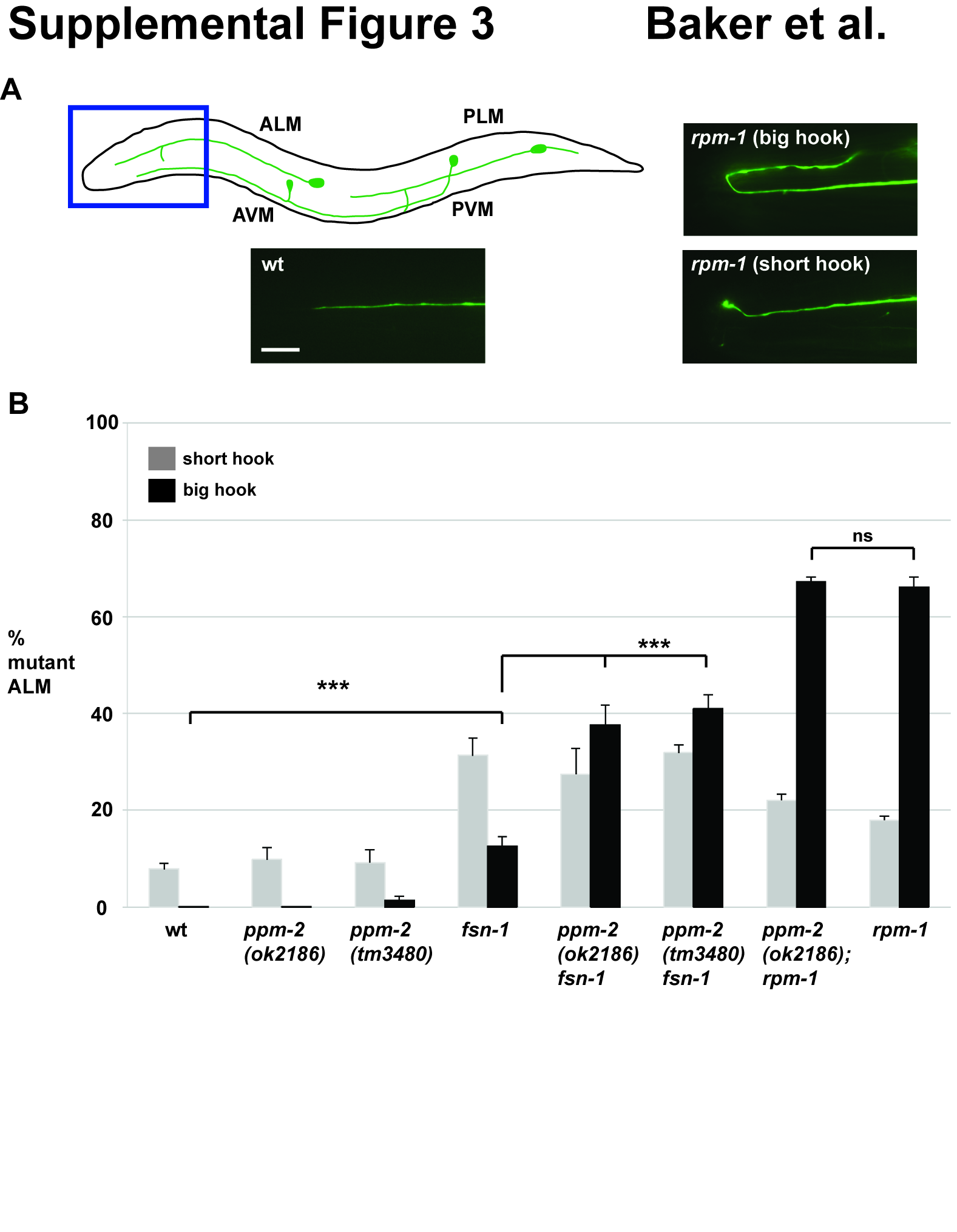

Supplement: Figure S3 — ppm-2 regulates axon termination in the ALM mechanosensory neurons. The axons of ALM mechanosensory neurons were visualized using the transgene muIs32 (Pmec-7GFP). (A) Epifluorescent microscopy was used to visualize the ALM axon in wild-type or rpm-1-/- mutants. The images shown correspond to the boxed region of the diagram. Note that in rpm-1-/- mutants two types of axon termination defects are visible: 1) more severe big hooks in which the axon overextends and hooks to the posterior of animal (top panel), and 2) less severe short hooks in which the axon overextends more modestly, and does not extend towards the posterior (lower panel). (B) Quantitation of specific, short hook (gray) or big hook (black), axon termination defects in the ALM mechanosensory neurons of the indicated genotypes. Averages are shown for data collected from 5–8 independent counts of 20–30 ALM neurons from adult worms grown at 23°C. Error bars represent the standard error of the mean, and significance was determined using an unpaired t-test. ***p<0.001 and ns = not significant. (TIF) [file pgen.1004297.s003.tif]

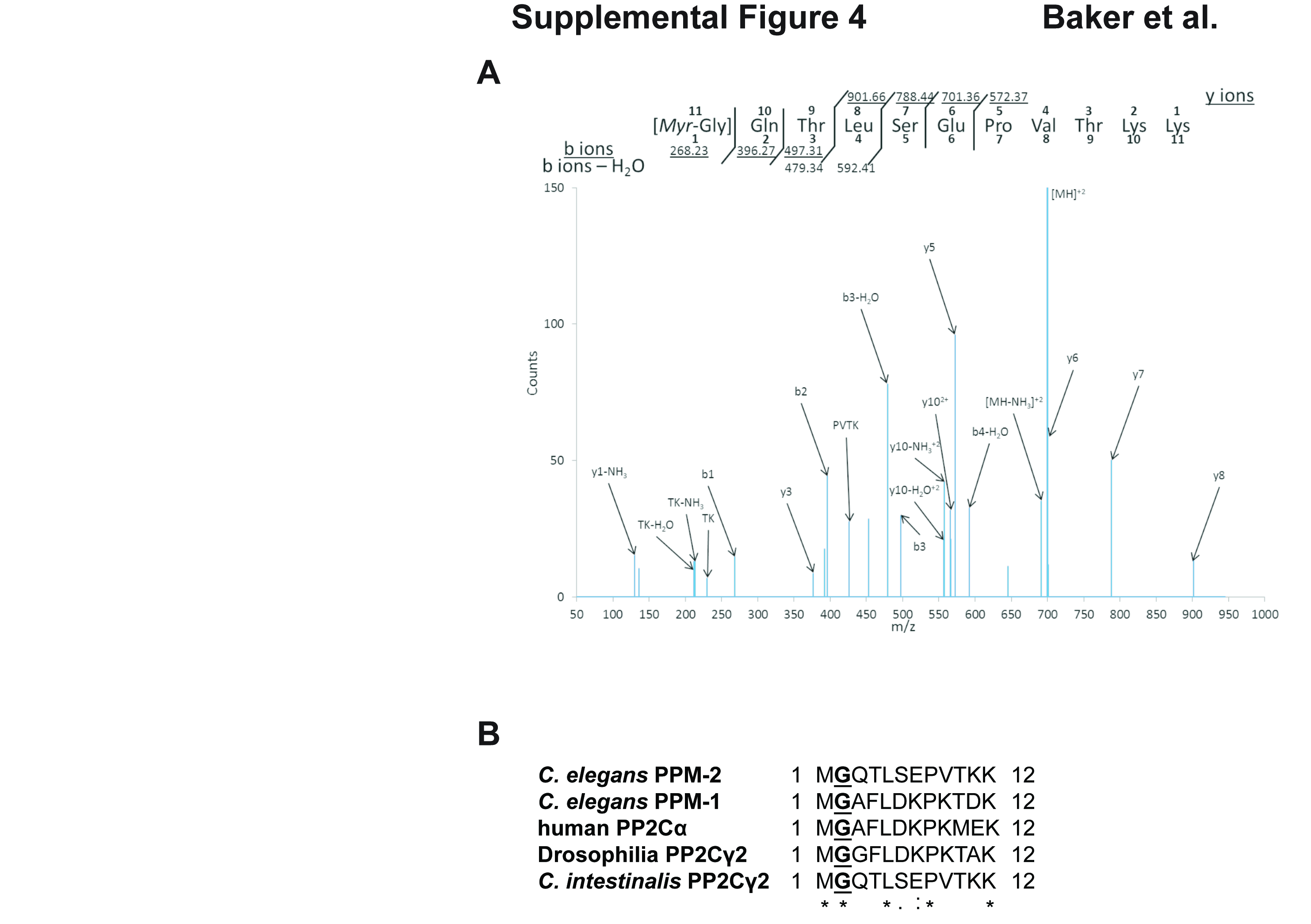

Supplement: Figure S4 — N-myristoylation of PPM-2. (A) Shown is a mass spectrum of the PPM-2 N-terminus peptide. Note that the size of ions b1, b2, b3 and b4 are shifted in size by 210 Da indicating that this PPM-2 peptide is N-myristoylated. (B) PPM-2 shares a conserved N-myristoylation site (G2) with several other indicated PP2C family phosphatases. Sequence analysis was done using EMBL-EBI ClustalW2. * (asterisk) indicates positions that are identical, (colon) indicates residues that show conservation between strongly similar amino acids, and. (period) indicates conservation between weakly similar amino acids. (TIF) [file pgen.1004297.s004.tif]
