# Supplementary material for: RPM-1 Uses Both Ubiquitin Ligase and Phosphatase-Based Mechanisms to Regulate DLK-1 during Neuronal Development
Source: PLoS Genet. 2014 May 8;10(5):e1004297. doi: 10.1371/journal.pgen.1004297 (PMC4014440; doi:10.1371/journal.pgen.1004297)
Supplement: Table S1 — A list of transgenic C. elegans strains used in this study. * Alleles used are ppm-2(ok2186), fsn-1 (gk429), dlk-1 (ju476), and rpm-1(ju44). ∧ Strains were constructed by injection of plasmid DNA, unless noted. (DOCX) [file pgen.1004297.s005.docx]

**Supplemental Table 1**

| **Reference Strain** | **Transgene** | **Figure** | **Injection Conditions^** | **Genotype*** |
| --- | --- | --- | --- | --- |
| XMN536 | *bggEx18* | 1A | P*_rpm-1_*RPM-1::GFP (pCZ161) (25ng/µL) | *rpm-1* |
| XMN537 | *bggEx85* | 1A | P*_rpm-1_*RPM-1::GFP (pCZ161) (25ng/µL);  P*_rgef-1_*FLAG::PPM-2 (pBG-GY135) (25ng/µL) | *ppm-2; rpm-1* |
|  |  | 1B,C | P*_ppm-2_*GFP (5ng/µL duplex PCR Product) | N2 |
| XMN508 | *bggEx102* | 1D,E | P*_ppm-2_*GFP (5ng/µL duplex PCR Product);  P*_mec-7_*mCherry (pBA182) (20ng/µL) | N2 |
|  |  | 3A | P*_mec-7_*PPM-2 (pBG-GY121) (5ng/µL) | *ppm-2 fsn-1; muIs32* |
|  |  | 3A | P*_mec-7_*PPM-2 *D59N* (pBG-GY204) (5ng/µL) | *ppm-2 fsn-1; muIs32* |
|  |  | 3A | P*_mec-7_*PPM-2 *G2A* (pBG-GY226) (5ng/µL) | *ppm-2 fsn-1; muIs32* |
|  |  | 3B | P*_mec-7_*PPM-2 (pBG-GY121) (5ng/µL) | *rpm-1; muIs32* |
|  |  | 3B | P*_mec-7_*PPM-2 *D59N* (pBG-GY204) (5ng/µL) | *rpm-1; muIs32* |
|  |  | 4B | P*_rgef-1_*DLK-1 (pBG-57) (2.5ng/µL PCR Product) | *muIs32* |
|  |  | 4B | P*_rgef-1_*DLK-1 (pBG-57) (2.5ng/µL PCR Product)  P*_mec-7_*PPM-2 (pBG-GY121) (5ng/µL) | *muIs32* |
|  |  | 4B | P*_rgef-1_*DLK-1 (pBG-57) (5ng/µL PCR Product);  P*_mec-7_*PPM-2 *D59N* (pBG-GY204) (5ng/µL) | *muIs32* |
|  |  | 4B | P*_rgef-1_*MKK-4 (pBG-GY310) (5ng/µL PCR Product) | *muIs32* |
|  |  | 4B | P*_rgef-1_*MKK-4 (pBG-GY310) (5ng/µL PCR Product);  P*_mec-7_*PPM-2 (pBG-GY204) (5ng/µL) | *muIs32* |
| XMN350 | *bggEx79* | 5A | P*_rgef-1_*FLAG::DLK-1 *K162R* (pBG-86) (10ng/µL PCR Product) | *dlk-1* |
| XMN352 | *bggEx81* | 5A | P*_rgef-1_*PPM-2::GFP *D59N* (pBG-GY313) (40ng/µL) | *ppm-2* |
| XMN429 | *bggEx96* | 5A | P*_rgef-1_*FLAG::DLK-1 *K162R* (pBG-86) (20ng/µL PCR Product)  P*_rgef-1_*PPM-2::GFP (pBG-GY312) (20ng/µL) | *dlk-1; ppm-2* |
| XMN430 | *bggEx97* | 5A | P*_rgef-1_*FLAG::DLK-1 *K162R* (pBG-86) (20ng/µL PCR Product)  P*_rgef-1_*PPM-2::GFP *D59N* (pBG-GY313) (40ng/µL) | *dlk-1; ppm-2* |
| XMN432 | *bggEx98* | 5B | P*_rgef-1_*FLAG::DLK-1 *K162R* (pBG-86) (20ng/µL PCR Product)  P*_rgef-1_*PPM-2::GFP *R185A* (pBG-GY377) (40ng/µL) | *dlk-1; ppm-2* |
| XMN538 | *bggEx104* | 5B | P*_rgef-1_*FLAG::DLK-1 *K162R* (pBG-86) (20ng/µL)  P*_rgef-1_*PPM-2::GFP (pBG-GY312) (20ng/µL) | *dlk-1; ppm-2* |
| XMN539 | *bggEx105* | 5B | P*_mec-7_*PPM-2::GFP *R185A* (pBG-GY377) (40ng/µL) | *ppm-2* |
| XMN423 | *bggEx89* | 5D | P*_rgef-1_*PPM-2::GFP (pBG-GY312) (5ng/µL) | *ppm-2* |
| XMN540 | *bggEx106* | 5D | P*_rgef-1_*PPM-2::GFP *D59N* (pBG-GY313)(5ng/µL) | *ppm-2* |
| XMN541 | *bggEx107* | 5E | P*_rgef-1_*PPM-2::GFP (pBG-GY312) (20ng/µL)  P*_rgef-1_*mCherry (pBG-GY371) (20ng/µL PCR Product) | *ppm-2* |
| XMN542 | *bggEx108* | 5E | P*_rgef-1_*PPM-2::GFP (pBG-GY312) (20ng/µL)  P*_rgef-1_*FLAG::DLK-1 *K162R* (pBG-86) (20ng/µL PCR Product) | *dlk-1; ppm-2* |
|  |  | 6A | P*_rgef-1_*DLK-1 (pBG-57) (2.5ng/µL PCR Product) | *muIs32* |
|  |  | 6A | P*_rgef-1_*DLK-1 (pBG-57) (2.5ng/µL PCR Product)  P*_mec-7_*PPM-2 (pBG-GY121) (5ng/µL) | *muIs32* |
|  |  | 6A | P*_rgef-1_*DLK-1 *S874E S878E* (pBG-144) (2.5ng/µL PCR Product) | *muIs32* |
|  |  | 6A | P*_rgef-1_*DLK-1 *S874E S878E* (pBG-144) (2.5ng/µL PCR Product)  P*_mec-7_*PPM-2 (pBG-GY121) (5ng/µL) | *muIs32* |
|  |  | 6A | P*_rgef-1_*DLK-1 *S874E* (pBG-158) (2.5ng/µL PCR Product) | *muIs32* |
|  |  | 6A | P*_rgef-1_*DLK-1 *S878E* (pBG-159) (2.5ng/µL PCR Product) | *muIs32* |
|  |  | 6A | P*_rgef-1_*DLK-1 *S874E* (pBG-158) (2.5ng/µL PCR Product)  P*_mec-7_*PPM-2 (pBG-GY121) (5ng/µL) | *muIs32* |
|  |  | 6A | P*_rgef-1_*DLK-1 *S878E* (pBG-159) (2.5ng/µL PCR Product)  P*_mec-7_*PPM-2 (pBG-GY121) (5ng/µL) | *muIs32* |
| XMN543 | *bggEx109* | 6B | P*_rgef-1_*FLAG::DLK-1L *K162R* (pBG-86) (20ng/µL PCR Product)  P*_rgef-1_*GFP::DLK-1S (pBG-GY477) (20ng/µL PCR Product) | N2 |
| XMN544 | *bggEx110* | 6B | P*_rgef-1_*FLAG::DLK-1L *K162R* (pBG-86) (20ng/µL PCR Product)  P*_rgef-1_*GFP::DLK-1S (pBG-GY477) (20ng/µL PCR Product) | *ppm-2* |
| XMN364 | *bggEx83* | 8A | P*_unc-25_*PPM-2::GFP (pBG-GY336) (10ng/µL) | *ppm-2* |
| XMN384 | *bggEx88* | 8B | P*_unc-25_*PPM-2::GFP (pBG-GY336) (10ng/µL)  P*_unc-25_*SNB-1::dsRed (pBG-59) (10ng/µL) | *ppm-2* |
